# SOX2 is essential for astrocyte maturation and its deletion leads to hyperactive behavior in mice

**DOI:** 10.1016/j.celrep.2022.111842

**Published:** 2022-12-20

**Authors:** Yan Wang, Sheng Zhang, Zhaohui Lan, Vui Doan, Bokyung Kim, Sihan Liu, Meina Zhu, Vanessa L. Hull, Sami Rihani, Chun-Li Zhang, John A. Gray, Fuzheng Guo

**Affiliations:** 1 Institute for Pediatric Regenerative Medicine, Shriners Hospitals for Children Northern California, Sacramento, CA 95817, USA; 2 Department of Neurology, School of Medicine, University of California, Davis, Davis, CA 95817, USA; 3 Department of Molecular Biology and Hamon Center for Regenerative Science and Medicine, University of Texas Southwestern Medical Center, Dallas, TX 75390, USA; 4 Center for Neuroscience, University of California, Davis, Davis, CA 95618, USA; 5 Present address: Department of Neurobiology, School of Basic Medical Sciences, Southern Medical University, Guangzhou 510515, China; 6 Present address: Bio-X Institutes, Brain Science and Technology Research Center, Shanghai Jiao Tong University, Shanghai 200240, China; 7 These authors contributed equally; 8 Lead contact

## Abstract

Children with *SOX2* deficiency develop ocular disorders and extra-ocular CNS anomalies. Animal data show that SOX2 is essential for retinal and neural stem cell development. In the CNS parenchyma, SOX2 is primarily expressed in astroglial and oligodendroglial cells. Here, we report a crucial role of astroglial SOX2 in postnatal brain development. Astroglial *Sox2*-deficient mice develop hyperactivity in locomotion and increased neuronal excitability in the corticostriatal circuit. *Sox2* deficiency inhibits postnatal astrocyte maturation molecularly, morphologically, and electrophysiologically without affecting astroglia proliferation. Mechanistically, SOX2 directly binds to a cohort of astrocytic signature and functional genes, the expression of which is significantly reduced in *Sox2*-deficient CNS and astrocytes. Consistently, *Sox2* deficiency remarkably reduces glutamate transporter expression and compromised astrocyte function of glutamate uptake. Our study provides insights into the cellular mechanisms underlying brain defects in children with *SOX2* mutations and suggests a link of astrocyte SOX2 with extra-ocular abnormalities in *SOX2*-mutant subjects.

## In brief

Wang et al. show an essential role of the stem cell factor SOX2 in astrocyte development and locomotion behavior in rodents through controlling a cohort of astrocytic signature and functional genes and regulating glutamate uptake. This work suggests cellular mechanisms underlying brain defects in children with *SOX2* mutations.

## Graphical Abstract

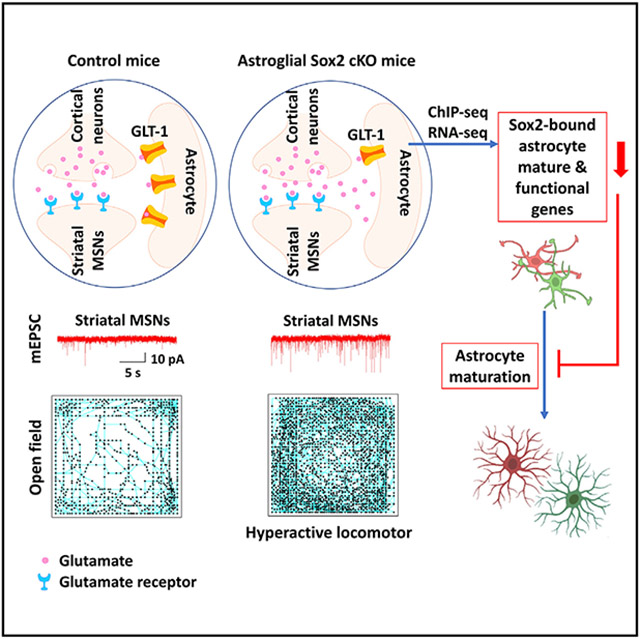

## INTRODUCTION

Individuals with heterozygous inactivation mutations of *SOX2* gene (SOX2 disorder)^1^ develop ocular defects and extra-ocular neurological impairments including cognition and motor dysfunctions and seizures. Previous animal studies establish that SOX2 plays essential roles in the maintenance and differentiation of retinal^2^ and neural stem cells,^3-5^ providing cellular mechanistic insights into ocular and brain defects of SOX2 disorder. Outside the neural stem cell (NSC) niche, SOX2 is expressed primarily in astroglial and oligodendroglial cells, and, to a much lesser extent, in cortical neurons, in the CNS parenchyma of mice^6-8^ and humans.^9^ Whether astroglial *SOX2*-deficiency contributes to neural defects and/or behavioral impairments remains elusive.

*SOX2* heterozygous mutations (~50% reduction) do not affect ocular^2,10^ or brain development^4^ in mice, but it does in humans.^1^ The human-mouse disparity in phenotypes associated with haploinsufficiency is likely due to a species difference in SOX2 sensitivity.^11^ Supporting this differential SOX2 sensitivity, reducing mouse SOX2 below 40% of the wild-type level indeed results in eye malformations^2^ and brain abnormalities.^4^ Due to the early embryonic lethality of *Sox2*-depleted mouse mutants, we used a *Sox2* conditional knockout (cKO) paradigm to interrogate the potential role of astroglial SOX2 in brain development and behavior. By employing astrocyte-specific *Cre-loxP* mouse genetics in combination with histological, molecular, and morphological assessment, unbiased bioinformatic analysis, electrophysiological recording, and behavioral test, we demonstrate that SOX2 is required for postnatal astrocyte maturation and essential for hyperactive locomotory function in mice. Astroglial *Sox2* deficiency results in enhanced excitability of neurons in the corticostriatal circuit, which has been shown to trigger hyperactive animal behavior. Our findings suggest that SOX2 dysfunction in postnatal astrocytes may contribute to brain anomalies and neurological defects in *SOX2*-deficient children.

## RESULTS

### Astrocytic *Sox2*-deficient mice and SOX2 deletion efficiency/specificity

We generated constitutive and inducible SOX2 cKO (referred to as *Sox2* cKO and *Sox2* icKO) mice using the Cre drivers *mGfap-Cre* and *Aldh1l1-CreER*^*T2*^, respectively (Figures S1A1 and S1B1). Histological assays demonstrated that SOX2 was deleted in >90% astrocytes labeled by brain lipid basic protein (BLBP) (gene: *Fabp7*)^12,13^ with no changes in Sox10^+^ oligodendroglial cells or NeuN^+^ neurons in different CNS regions (Figure S1) of P14 *Sox2* cKO mice and of P13 *Sox2* icKO mice that received TM (200 μg/g body weight) and OH-TM (100 μg/g body weight) at postnatal day 1 (P1)–P3, which is consistent with our previous analysis of EYFP-reporter-based astrocyte specificity.^14^

### Hyperactivity in the locomotory behavior of astrocytic *Sox2*-mutant mice

**Figure 1.**
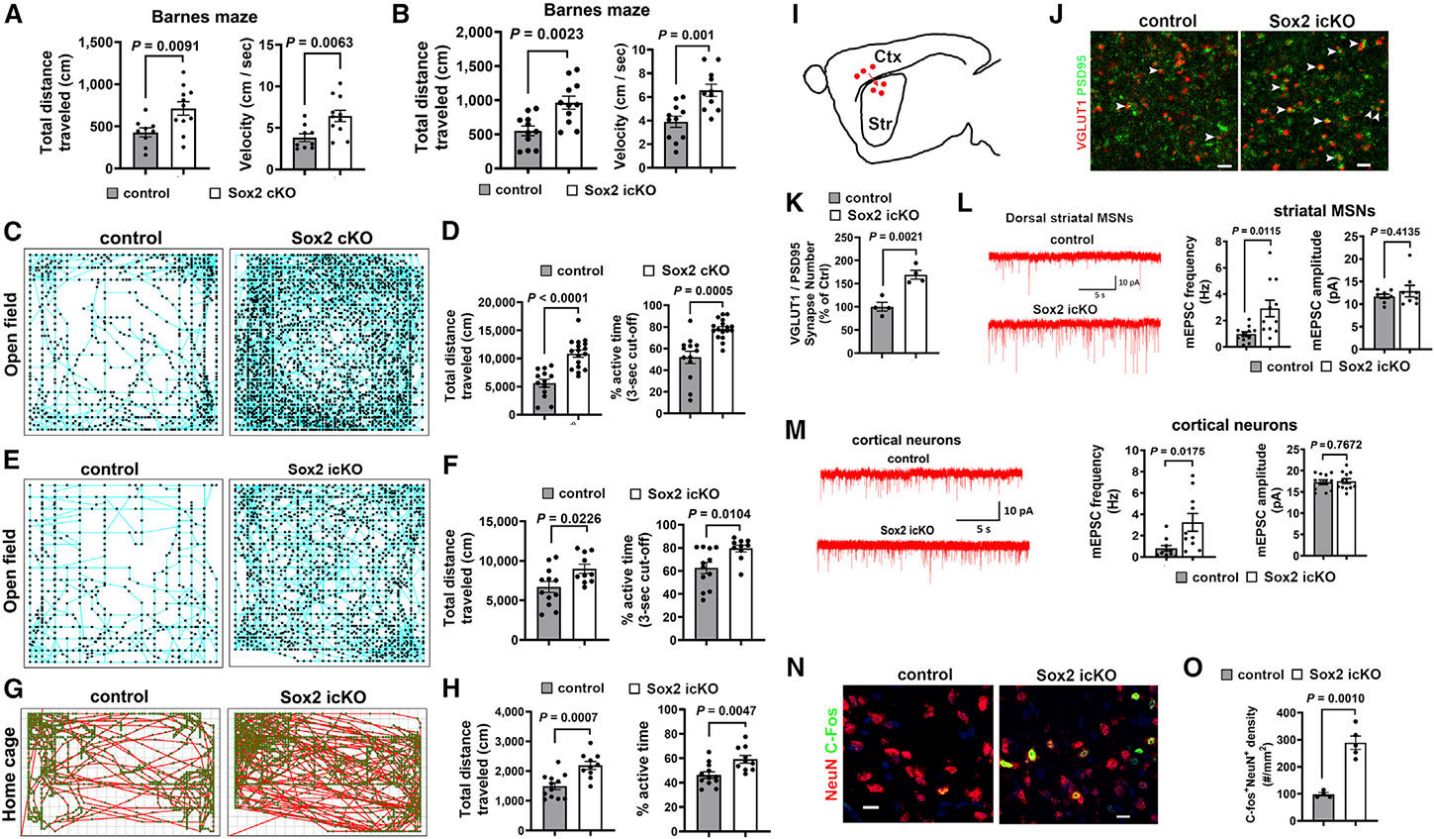
Astrocytic SOX2 depletion leads to hyperactive locomotor behavior and elevated neuronal excitability (A and B) Total distance and velocity of Barnes maze test of (A) *mGfap-Cre:Sox2*^*fl/fl*^ (n = 9 control, 11 Sox2 cKO) and (B) *Aldh1l1-CreER^T2^:Sox2^fl/fl^* (n = 11 control, 11 Sox2 icKO) mice. (C–F) Movement tracing and quantification of open field test of (C and D) *mGfap-Cre:Sox2^fl/fl^* (n = 13 control, 17 Sox2 cKO) and (E and F) *Aldh1l1-CreER^T2^:Sox2^fl/fl^* (n = 12 control, 10 Sox2 icKO) mice. (G and H) Movement tracing and quantification of home-cages test. n = 12 control, 10 Sox2 icKO. (I) Diagram showing cortical (Ctx) neuronal projections onto dorsal striatal (Str) medium spinal neurons (MSNs) in the corticostriatal circuitry. (J and K) Representative images and quantification of VGlut1 and PSD95 in the dorsal striatum. Arrowheads, VGlut1/PSD95 co-labeled puncta. n = 4 control, 4 Sox2 icKO. (L and M) Current tracing and quantification of miniature excitatory postsynaptic currents (mEPSCs) of dorsal Str MSNs (mEPSC frequency, n = 11 control, 11 Sox2 icKO; mEPSC amplitude, n = 8 control, 8 Sox2 icKO) and Ctx neurons (mEPSC frequency, n = 10 control, 10 Sox2 icKO; mEPSC amplitude, n = 12 control, 12 Sox2 icKO). (N and O) Representative images and density of C-fos^+^NeuN^+^ cells in the dorsal striatum. n = 4 control, 5 Sox2 icKO. Error bars indicate means ± SEM. Unpaired two-tailed Student’s t test was used for statistically analyzing two groups of data. Please see Table S6 for statistics. Animal ages: (A–K) 2 months, (L and M) P35 (tamoxifen P15/P16), and (N and O) P21 (tamoxifen P4–P9). n, biological replicates. Scale bars: (J) 1 μm and (N) 10 μm.

The potential behavioral outcome of mice with astrocytic *Sox2* mutation remains enigmatic. Barnes maze test (Figure S2A1) showed comparable ability of spatial learning and memory in adult *Sox2* cKO (Figure S2A2) and *Sox2* icKO (Figure S2A3) mice, but they exhibited a significant increase in total distance and velocity of locomotor (Figures 1A and 1B), indicating a hyperactive locomotory behavior. To confirm the hyperactivity, we employed the open-field test (Figures 1C and 1E), a classical paradigm used to assess locomotory activity,^15^ and found that *Sox2* cKO and *Sox2* icKO mice traveled significantly longer distances and were more active (Figures 1D and 1F) than their controls. We also found that *Sox2* icKO mice were hyperactive in the vertical dimension of the open field as evidenced by significantly more bouts of sudden jumps during test sessions (jumping bouts: 6.2 ± 2.7 control [Ctrl] [n = 5] versus 21.3 ± 3 *Sox2* icKO [n = 7], mean ± SEM, two-tailed Student’s t test, *t*_(10)_ = 3.545, p = 0.005) (Videos S1, Ctrl, and S2, *Sox2* icKO).

To further strengthen the conclusion, we used the home-cage test (Figure 1G) in a natural environment. *Sox2* icKO mice traveled longer distances and spent more time moving (Figure 1H) in the cages than Ctrl mice. However, Rotarod (Figure S2B) and CatWalk (Figure S2C) revealed a comparable function of motor coordination and walking gait. A three-chamber test (Figure S2D1) showed no differences in sociability (Figure S2D2) or social memory and novelty (Figure S2D3) of *Sox2* cKO mice. Together, these data demonstrate that *Sox2* mutations in astrocytes lead to hyperactive locomotion yet are dispensable for the development of motor skills and cognition in mice.

### Elevated excitability of neurons in the corticostriatal circuit of astrocytic *Sox2*-mutant mice

The spontaneous hyperactivity suggests a neuronal perturbation in the motor circuitry. The corticostriatal circuit plays an essential role in motor control.^16^ Activating striatal medium spinal neurons (MSNs), the most abundant cell type in the striatum,^17^ elicits hyperactive locomotion in mice.^18^ Since the cortical projection onto striatal MSNs (Figure 1I) is glutamatergic, we employed VGlut1 as presynaptic and PSD95 as postsynaptic markers to quantify the synaptic co-localization in high-power confocal images according to the previously validated protocol.^19^ Our data showed an increased density of co-localized puncta in the dorsal striatum of *Sox2* icKO mice (Figures 1J and 1K). Next, we patch clamped dorsal striatal MSNs in acutely prepared sagittal brain slices and recorded miniature excitatory postsynaptic currents (mEPSCs) and found that the frequency, but not the amplitude, was increased by ~3-fold higher in *Sox2* icKO mice (Figure 1L), suggesting an increased number of functional synaptic inputs onto striatal MSNs. We also found that the frequency of mEPSCs was significantly increased in the cortical layer V neurons projecting onto striatal MSNs (Figure 1M). c-Fos is an immediate-early gene activated in neurons following membrane depolarization, and its expression is widely used as a marker for neuronal activation.^20^ The density of c-Fos^+^ striatal MSNs was significantly increased in astroglial *Sox2*-deficient mice (Figures 1N and 1O). Collectively, our data suggest that astrocytic *Sox2* mutation results in elevated excitability of striatal MSNs, which has been shown to mechanistically cause hyperactive locomotory behavior in mice.^18^

### Perturbed astrocyte maturation and normal astrocyte proliferation in *Sox2*-mutant mice

Little is known about the cellular anomalies in the brain of children with *SOX2* mutations. We speculate that *Sox2* mutations affect postnatal astrocyte development. Astrocyte maturation occurs primarily during first-month postnatal ages in mice. *Sox2* KO reduced the CNS expression of signature genes enriched in astrocytes (for instance, *Gfap, Id3, Id4, Wnt7a*) in both *Sox2* cKO and *Sox2* icKO (Figure S3A1-S3A4 and S3B1-S3B2) mice, suggesting that SOX2 regulates molecular maturation of postnatal astrocytes. Astrocytes expand their population via local proliferation of BLBP^+^ immature astrocytes in the murine cortex, and this occurs predominantly by the first 2 postnatal weeks.^13,21^ *Sox2* icKO did not affect the densities of total astrocytes nor proliferating astrocytes in the cerebral cortex at P13 (Figure S3B3).

**Figure 2.**
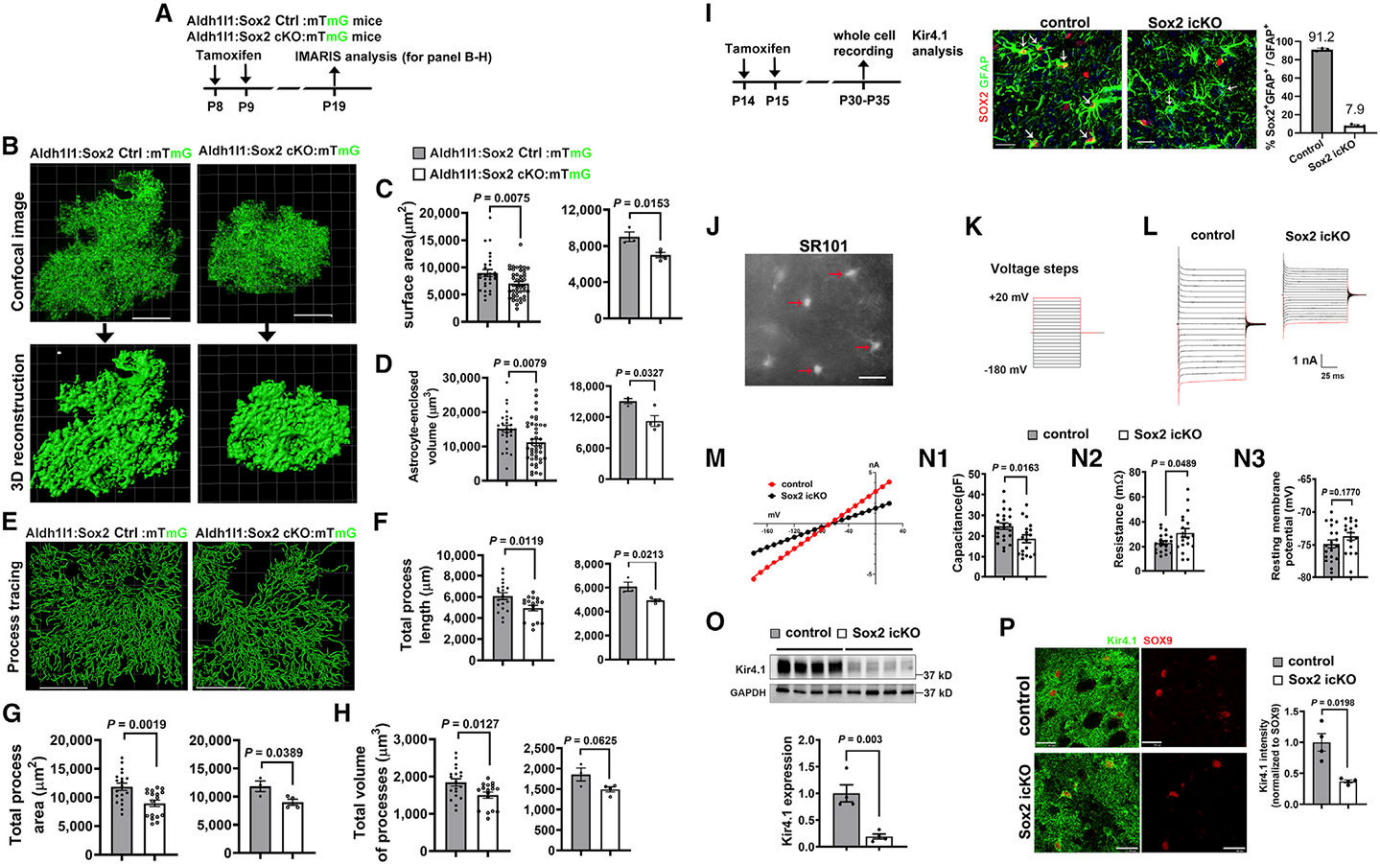
Astrocytic SOX2 depletion alters morphological and physiological properties of astrocytes (A) Experimental design for (B)–(H). (B) Representative images of maximal projection and 3D reconstruction (IMARIS) of mG^+^ astrocytes. (C and D) Surface area and enclosed volume of mG^+^ astrocytes. Graphed as means of each astrocyte in *Aldh1l1-CreER^T2^:Sox2^fl/fl^* mice (left, n = 28 control, 43 Sox2 icKO) and mouse (right, n = 3 control, 4 Sox2 icKO). (E–H) Automatic tracing of mG^+^ astrocyte processes by the filament tool of IMARIS and quantifications. Graphed as means of each astrocyte (left, n = 19 control, 17 Sox2 icKO) and mouse (right, n = 3 control, 4 Sox2 icKO). (I) Left, experimental design for (J)–(P); right, immunofluorescence of SOX2 and GFAP and quantification. n = 4 control, 4 Sox2 icKO. (J) Ctx astrocytes visualized by SR101 (arrows) for whole-cell recordings. (K) Voltage steps for astrocyte recording, from −180 to +20 mV with a step size of 10 mV. (L and M) Representative current tracing (L) and I-V curve (M) of one *Sox2*-deficient and one *Sox2*-intact astrocyte. (N1–N3) Quantification of cell capacitance (N1, n = 23 control, 18 Sox2 icKO), input resistance (N2, n = 20 control, 19 Sox2 icKO), and resting membrane potential (N3, n = 20 control, 17 Sox2 icKO) of Ctx astrocytes. (O) Western blot and quantification of Kir4.1 in the brain. n = 4 control, 4 Sox2 icKO. (P) Representative images and quantification of Kir4.1 intensity in the cortex. n = 4 control, 4 Sox2 icKO. Error bars indicate means ± SEM. Unpaired two-tailed Student’s t test was used for statistically analyzing two groups of data. Please see Table S6 for statistics. n, biological replicates. Scale bars, (B, E, I, J, and P) 20 μm.

Given that astrocytic fine processes are formed primarily during the third postnatal week in the murine cortex,^22^ we evaluated astrocyte morphological maturation at P19 by IMARIS analysis of membrane-bound GFP (mGFP)-labeled astrocytes and processes (Figures 2A, 2B, and 2E). Our data showed that SOX2 deletion decreased astrocytic surface area (Figure 2C) and volume (Figure 2D). Tracing mGFP^+^ processes of individual astrocytes (Figure 2E) showed a significant reduction in the total process length (Figure 2F), process surface area (Figure 2G), and process volume (Figure 2H) of *Sox2*-mutant astrocytes. Together, our analyses indicate that SOX2 is required for astrocyte maturation morphologically.

Next, we measured the electrophysiological properties of astrocytes at ~1 month (Figure 2I), when most astrocytes reach a mature state electrophysiologically, which displays a characteristic linear current/voltage relationship (I/V curve).^23,24^ Histological analysis confirmed ~90% deletion efficiency in cortical astrocytes (Figure 2I). We used the fluorescence dye SR101^25^ to visualize astrocytes in brain slices (Figure 2J). Applying stepped voltages (Figure 2K) yielded nearly linear current responses in *Sox2*-deficient and -intact astrocytes (linear I/V curve) (Figures 2L and 2M). We found that membrane capacitance of *Sox2*-deficient astrocytes was smaller than *Sox2*-intact ones (Figure 2N1), which is consistent with the smaller cell surface area and less complex morphology we documented for *Sox2*-deficient astrocytes. Furthermore, *Sox2*-deficient astrocytes displayed an increase in the membrane resistance (Figure 2N2) and a slightly (but not significantly; p > 0.05) more positive resting membrane potential (Figure 2N3). The passive conductance property of astrocytes is conferred mainly by the inwardly rectifying K^+^ channel Kir4.1.^26^ Consistently, we found that *Sox2* disruption remarkably reduced brain Kir4.1 expression at the protein (Figure 2O) and histological (Figure 2P) levels.

Taken together, our data suggest that SOX2 regulates postnatal astrocyte maturation at the molecular, morphological, and electrophysiological levels and is dispensable for astrocyte proliferation and population expansion.

### SOX2 targets a cohort of genes that are crucial for astrocyte maturation and function

**Figure 3.**
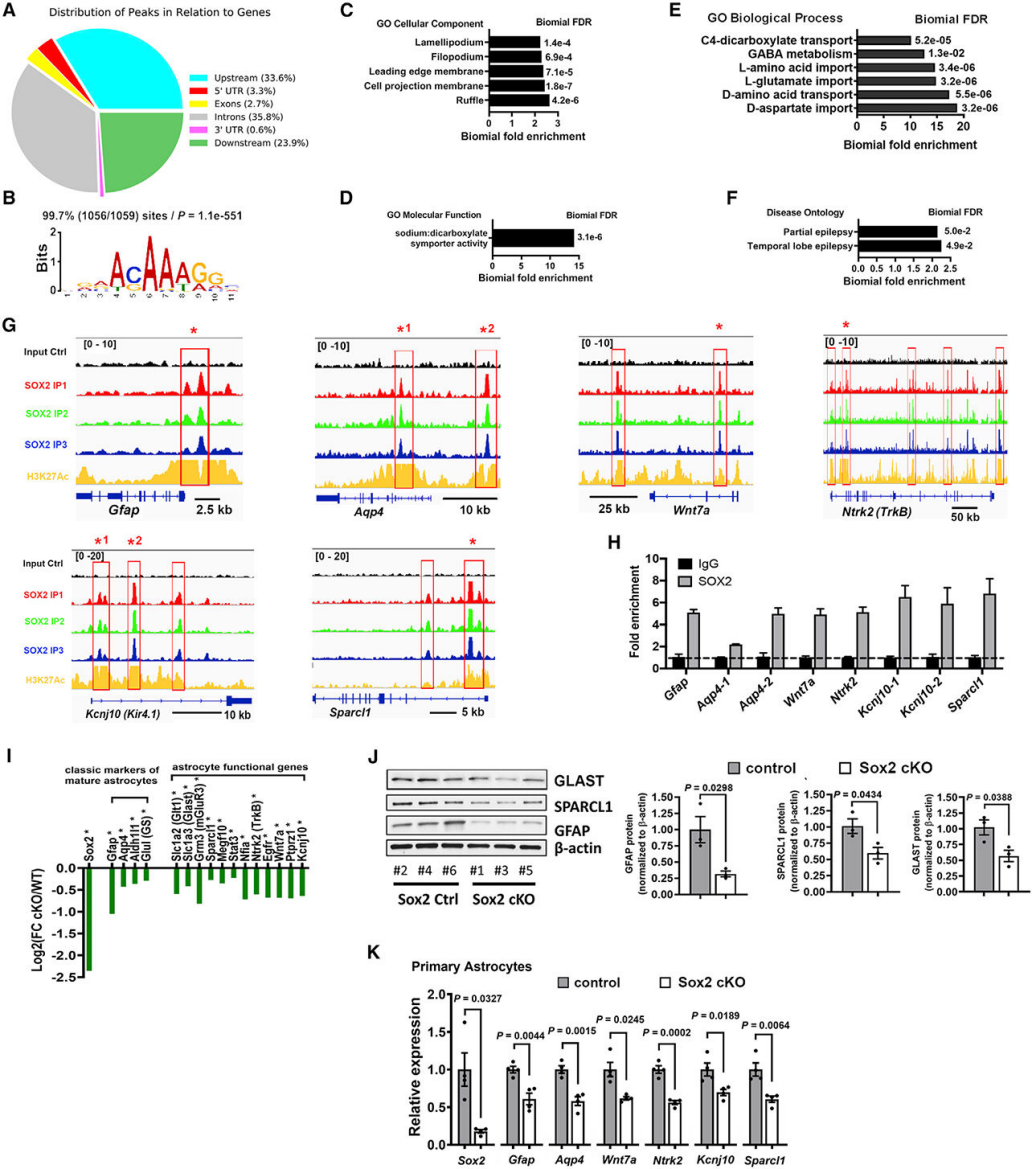
Genomic and transcriptomic profiling identifies SOX2-regulated genes and pathways (A) Genomic distribution of SOX2-bound peaks. (B) Top enriched motif CAAAG (Table S3) is present in 99.7% of the top 1,059 SOX2-bound sites (Table S2). (C–E) Significantly enriched GO terms among the top 1,059 SOX2-bound genomic regions. (F) Overrepresented Disease Ontology terms of the top 1,059 SOX2-bound genomic regions. (G) Genome browser views of SOX2 binding and H3K27Ac overlapping in the astrocytic genes *Gfap, Apq4, Wnt7a, Ntrk2, Kcnj10*, and *Sparcl1*. (H) ChIP-qPCR verification of SOX2 binding at the sites marked by stars in (G) in primary astrocytes cultured in serum-free medium. n = 3 biological replicates. (I) Representative downregulated genes identified by bulk RNA-seq. Asterisks indicate SOX2-bound genes (Table S1). (J) Western blot assay of representative downregulated DEGs in the brain of m*Gfap-Cre:Sox2^fl/fl^* mice. n = 3 control, 3 Sox2 cKO. (K) qRT-PCR assays for representative SOX2-bound genes in primary astrocytes. n = 4 control, 4 Sox2 cKO. n, biological replicates. Error bars indicate means ± SEM. Unpaired two-tailed Student’s t test was used for statistically analyzing two groups of data. Please see Table S6 for statistics.

We sought to identify SOX2-regulated target genes using our previously deposited datasets (GEO Database: GSE85213).^8^ 9,602 SOX2-bound sites were identified with false discovery rate (FDR) <0.05 (Table S1); these sites displayed a similar genome distribution pattern (Figure 3A) as we previously reported.^8^ Among the 9,602 SOX2-bound sites, 1,059 were identified confidently based on their peak fold enrichments being greater than 10, which were associated with 879 protein-coding and 37 microRNA-coding genes (Table S2). Motif analysis using MEME-ChIP^27^ demonstrated that nearly 100% of the binding sites (1,056 out of 1,059) contained the CAAAG sequence and its variants (Figure 3B; Table S3), the canonical SOX2 motif,^28^ indicating that these identified genomic regions are highly likely targeted by SOX2 with a much higher confidence than we identified previously.^8^

To provide insights into SOX2’s function, we annotated the 1,059 SOX2-bound sites using GREAT.^29^ The protein products of SOX2-bound genes were primarily located in the ruffle, cell projection, and leading-edge membranes (Figure 3C), which is essential for astrocyte process extension during morphological maturation.^30^ Amino acid (such as glutamate) transport activity was the top enriched GO term (Figures 3D and 3E; Table S4), which was further supported by Disease Ontology analysis showing that epilepsy, a neurological condition in which astrocytic regulation of glutamate homeostasis is compromised,^31^ was significantly enriched (Figure 3F). The SOX2-bound genes that are associated with glutamate import and epilepsy were identified as *Kcnj10* (Kir4.1), *Slc1a2* (GLT-1), and *Slc1a3* (GLAST). In addition, SOX2 also bound to other characteristic astrocyte genes, for example *Gfap, Apq4, Wnt7a, Ntrk2,* and *Sparcl1* (Figure 3G), among which *Ntrk2* has been shown to promote astrocyte morphological maturation both *in vivo* and in culture.^32^ Chromatin immunoprecipitation (ChIP)-qPCR verified SOX2 binding to the regulatory elements of these key astrocytic functional genes in primary astrocytes (Figures 3H and S4). Furthermore, these SOX2-bound regions were co-occupied by H3K27Ac (Figure 3G), an epigenetic marker of active enhancers,^33^ suggesting that SOX2 may activate their expression.

To determine the significance of SOX2’s genomic binding activity *in vivo,* we disrupted SOX2 and searched for transcriptomic changes in the CNS by RNA sequencing (RNA-seq). We identified 2,342 downregulated and 1,984 upregulated differentially expressed genes (DEGs) in *Sox2* cKO mice (Table S5). We intersected the 1,059 SOX2-bound sites identified by ChIP-seq with the DEGs identified by RNA-seq and found that 30% (263/879) of SOX2-bound protein-coding genes were dysregulated, among which the majority (208/263, 79%) were downregulated, whereas only 21% were upregulated (Table S2), in *Sox2* cKO mice, suggesting that SOX2’s genomic binding may act mainly as a transcriptional activator for its target genes in astrocytes.

RNA-seq identified significant reduction in the genes encoding canonical markers of astrocytes (for example, *Gfap, Aqp4, Aldh1l1*) (Figure 3I) and astrocytic functional genes, for instance glutamate transport genes *Slc1a2* and *Slc1a3,*^34^ astrocytic morphogenesis-promoting gene *Ntrk2*,^32^ and K^+^-buffering gene *Kcnj10*^26^ (Figure 3I). Western blot confirmed the decrease in the protein level of, for example, GFAP and SPARCL1 (Figure 3J) and Kir4.1 (cf. Figures 2O and 2P) in the brain. Importantly, qRT-PCR showed that the expression levels of these key genes were significantly decreased in primary astrocytes purified from *Sox2* cKO mice compared with Ctrls (Figures 3K and S4), which links the downregulated genes discovered in whole-tissue RNA-seq directly to astrocytes. These data indicate that astroglial *Sox2* deficiency elicits a comprehensive molecular change in the postnatal CNS and astrocytes.

### SOX2 deficiency compromises astrocytic glutamate uptake function

**Figure 4.**
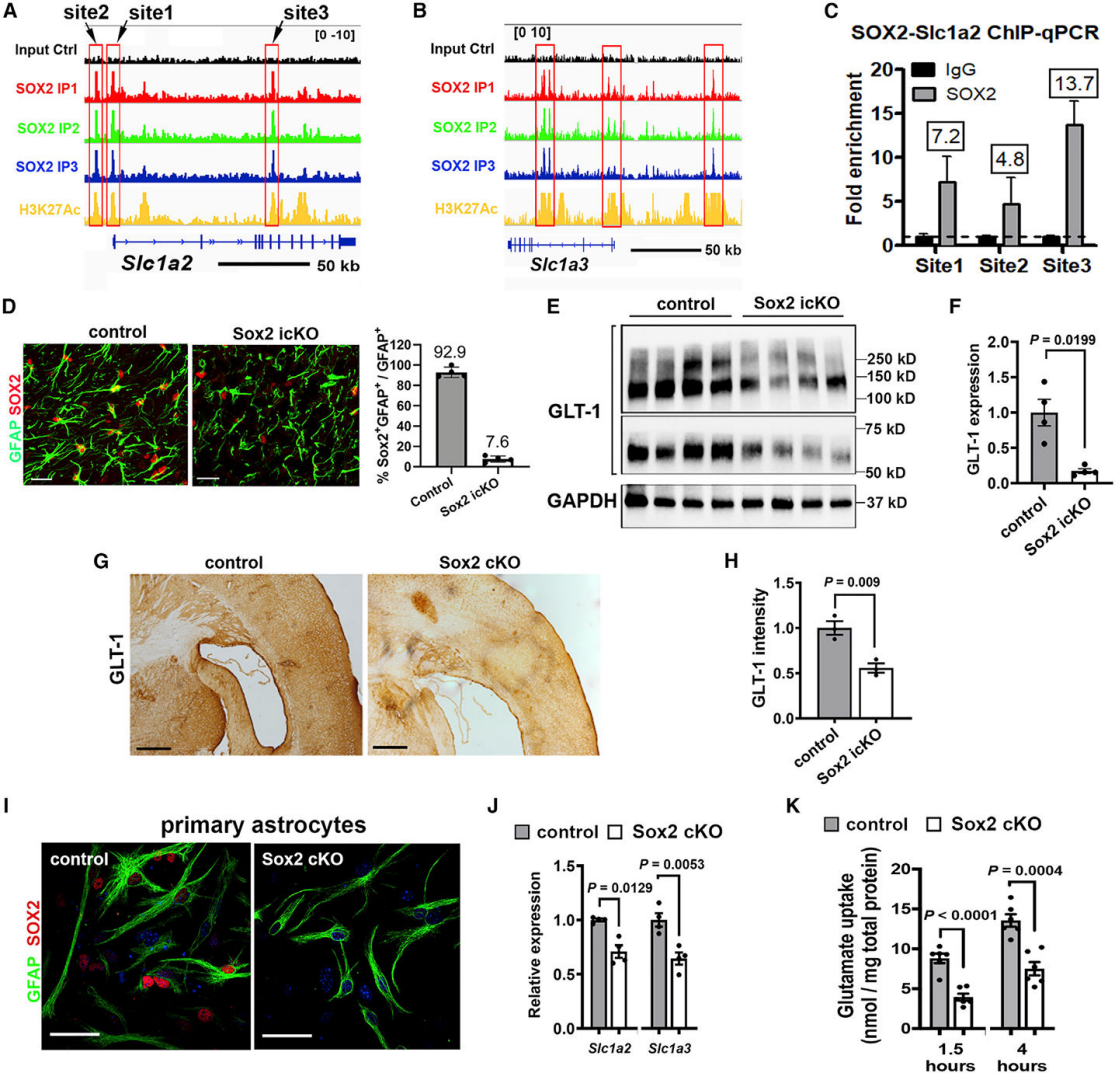
Downregulation of glutamate transporters and impaired glutamate uptake of Sox2-deficient astrocytes (A and B) Genome browser view of SOX2-bound sites and co-occupancy by H3K27Ac in in *Slc1a2* and *Slc1a3* genes. (C) ChIP-qPCR verification of SOX2 binding to *Slc1a2* in primary astrocytes. n = 3 biological replicates. (D) Representative images and quantification of SOX2/GFAP in the cortex of *Aldh1l1-CreR^T2^:Sox2^fl/fl^* mice. n = 4 control, 4 Sox2 icKO. (E and F) Western blot images and quantification of GLT-1 in the brain. 62 (monomer), 125 (dimer), and 250 kD (tetramer) of GLT-1. n = 4 control, 4 Sox2 icKO. (G and H) Representative DAB histological images and quantification of GLT1 in the brain. n = 3 control, 3 Sox2 icKO. (I) Representative immunofluorescence of GFAP and SOX2 in serum-free primary astrocytes (14 days *in vitro* [DIV14]) (Figure S4) isolated from *mGfap-Cre:Sox2^fl/fl^* mice. (J) qRT-PCR assay for *Slc1a2* and *Slc1a3* in primary astrocytes. n = 4 control, 4 Sox2 cKO. (K) Glutamate uptake of primary astrocytes at 1.5 and 4 h after glutamate incubation. n = 6 control, 6 Sox2 cKO. (D–H) P21 *Aldh1l1-CreR^T2^:Sox2^fl/fl^* mice (tamoxifen injection at P4–P9). n, biological replicates. Scale bars, (D) 20 μm, (G) 200 μm, and (I) 50 μm.

The overrepresented GO terms (Figures 3D-3F) suggest that SOX2 regulates glutamate transporter expression and glutamate uptake function of astrocytes. In the CNS, GLT-1 (*Slc1a2*), and to a lesser extent, GLAST (*Slc1a3*), is the major transporter that takes up >90% of glutamate and maintains optimal extracellular glutamate levels, thus preventing neuronal hyperactivation.^34^ SOX2 binds to multiple *cis* elements of the mouse *Slc1a2* and *Slc1a3* genes (Table S1). These SOX2-bound sites were co-occupied by the epigenetic marker of active enhancers H3K27Ac (Figures 4A and 4B). ChIP-qPCR confirmed the physical binding of SOX2 at the *cis* elements of *Slc1a2* in purified primary astrocytes (Figures 4A, 4C, and S4). To gain insights into SOX2-GLT-1 regulation *in vivo,* we disrupted SOX2 in astrocytes (Figure 4D) and assessed brain GLT-1 (Figure 4E). Our results demonstrated a ~5-fold reduction in GLT-1 protein level in *Sox2* icKO brain (Figure 4F). The diminution was further confirmed by histological staining (Figures 4G and 4H). We showed a significantly decreased expression of GLT-1 and GLAST (Figure 4I) in *Sox2*-deficient primary astrocytes (Figure 4K), suggesting that *Sox2*-deficient astrocytes may have impaired ability of extracellular glutamate uptake. To test this hypothesis, we performed a glutamate uptake experiment by incubating DIV14 GFAP^+^ mature astrocytes with added glutamate. Sox2-deficient astrocytes displayed reduced ability of glutamate uptake from the medium at 1.5 and 4 h (Figure 4K). Collectively, our data suggest that SOX2 regulates the expression of astrocytic glutamate transporters and that SOX2 deficiency compromises the astrocytic function of extracellular glutamate removal.

## DISCUSSION

*SOX2* deficiency in children leads to ocular disorders and extra-ocular neurological dysfunction. It appears that SOX2 expression in various populations of neural cells controls different behavioral outputs. Mice with SOX2 KO in early neural precursor cells exhibit seizures and motor dysfunction.^4^ SOX2 KO in Nestin^+^ NSCs causes a more restricted neurogenic defect and a progressive loss of the hippocampus,^5^ suggesting that SOX2 in NSCs may be responsible for cognitive disabilities given the importance of the hippocampus in cognition. Recent studies demonstrated that mice with SOX2 deletion in oligodendroglial cells exhibit CNS hypomyelination and develop ataxia and motor coordination defects.^6,35^ The current finding suggests that SOX2 functional deficiency in astroglial lineage may also contributes to the brain anomalies and neurological impairments in *SOX2*-mutated children.

The dispensability of SOX2 in astrocyte proliferation and population expansion (Figure S3B3) is quite surprising given that local proliferation is the main mechanism for astrocyte population expansion in the postnatal murine brain.^13,36^ SOX2 plays an essential role in the proliferation of NSCs and neural precursor cells.^4,5^ The molecular mechanisms underlying postnatal astrocyte proliferation remain elusive but seem to be SOX2 independent. Alternatively, the reported redundancy between SOX2 and SOX1/3^37^ may account for normal proliferation of *Sox2*-deficient astrocytes. We reported that SOX2 disruption in adult quiescent astrocytes inhibits their proliferation in response to traumatic brain injury. These data suggest that SOX2 regulation of astrocyte proliferation is context dependent (development versus injury). Interestingly, SOX2 persists in quiescent astrocytes in the adult CNS^7^; more than 85% of SOX2^+^ cells are astrocytes in the cortex (data not shown). It seems that SOX2 may control astrocyte quiescence, a prerequisite for their normal functions under homeostatic conditions. Our previous data show that SOX2 is upregulated in both proliferative and non-proliferative reactive astrocytes in response to inflammatory demyelinating injury,^7^ suggesting that SOX2 may additionally regulate astrocyte activation and/or function without interfering with its proliferation even under injury conditions. The role of SOX2 in astrocyte activation and astrocyte function remains enigmatic and warrants further study. Our genetic model (*Sox2* icKO) provides a valuable tool to probe these open questions.

In addition to molecular maturation, SOX2 is also necessary for morphological maturation of astrocytes in the CNS. By utilizing a conditional, membrane-bound reporter mGFP and morphological analysis, we found that *Sox2*-deficient astrocytes develop a less complex process network and surface area and occupy a smaller domain volume. The less complex morphology is congruent with the decreased capacitance of *Sox2*-deficient astrocytes, which is directly proportional to the membrane surface area.^38^ The increased membrane resistance of *Sox2*-deficient astrocytes is likely due to reduced ion channel expression of Kir4.1, which is the major channel conferring astrocyte membrane conductance.^26^ Mild reduction in astrocyte Kir4.1 (by ~30%) is sufficient to enhance the excitability of striatal MSNs and elicit motor deficits in a mouse model of Huntington’s disease.^39^ Strikingly, we found that *Sox2* disruption results in a >2-fold reduction of Kir4.1 expression in the brain. It is plausible that *Sox2*-disruption-elicited Kir4.1 downregulation may contribute, at least in part, to the enhanced excitability of MSNs we observed.

Unbiased GO analysis of SOX2-binding regions points to a key role of SOX2 in regulating genes related to glutamate uptake. Astrocytes take up the majority extracellular glutamate after neuronal depolarization to avoid network overactivation and play an important role in maintaining a balance of excitation and inhibition (E/I balance).^31^ Both GLT-1 and Kir4.1 are functionally essential for extracellular glutamate uptake by astrocytes.^31,40^ Our data show that GLT-1 is also targeted by SOX2 in astrocytes and that SOX2 disruption results in remarkable GLT-1 downregulation. Consistently, our functional analysis demonstrates that *Sox2*-deficient astrocytes display significantly compromised capability to uptake extracellular glutamate. These data suggest that SOX2 regulates functional maturation of postnatal astrocytes presumably through directly targeting glutamate transporters, particularly GLT-1.

Taken together, using astroglial-specific SOX2 KO mutants and a variety of approaches, we report that SOX2 plays an important role in astrocyte developmental maturation and controls motor hyperactivity. Together with the recent report that SOX2 is required for CNS myelination and motor skill development,^6,35,37,41^ our data suggest that glial SOX2 coordinates postnatal brain development and regulates animal motor skill and motor activity, thus providing insights into explaining the brain abnormalities of *SOX2*-deficient children.

### Limitations of the study

There are several limitations of the study. The current study did not consider astrocyte regional heterogeneity, which underlies astrocyte functional diversity. Our neuron recording showed an increase in frequency (but not amplitude) of mEPSCs, suggesting that Sox2-deficient astrocytes affect neuron transmission by a presynaptic mechanism. Further studies are needed to prove this hypothesis. Some key dysregulated signature/functional genes of astrocytes were individually verified by PCR; however, single-cell RNA-seq (scRNA-seq) of brain cells and/or ChIP-seq of astrocytes are needed to screen SOX2-regulated transcriptomic/genomic profiles. The purity of astrocyte culture in serum-free condition assessed by GFAP expression may be underestimated since some resting astrocytes in rodent brain do not express GFAP under physiological condition.

## STAR★METHODS

### RESOURCE AVAILABILITY

#### Lead contact

Further information and requests for resources and reagents should be directed to and will be fulfilled by the lead contact, Fuzheng Guo (fzguo@ucdavis.edu).

#### Materials availability

All reagents will be made available by the lead contact author after completion of a Materials Transfer Agreement.

#### Data and code availability

- The original data within the paper will be available from the lead contact upon request. RNA sequencing and ChIP sequencing data have been deposited at NCBI GEO and are publicly available. Accession numbers are listed in the key resources table. RNA-seq, Database: GSE182032 (Guo lab); ChIP-seq, Database: GSE85213 (Zhang lab); ChIP-seq, Database: GSE96539 (Berninger Lab).
- This paper does not report original code.
- Any additional information in this paper is available from the lead contact upon request.

### EXPERIMENTAL MODEL AND SUBJECT DETAILS

#### Transgenic mice - Cre lines, and reporter lines

All mice were housed at 12 h light/dark cycle with free access to food and drink, and both males and females were used in this study. All transgenic mice were maintained on a C57BL/6 background and approved by Institutional Animal Care and Use Committee at the University of California, Davis. B6; FVB-Tg(Aldh1l1-cre/ERT2)1Khakh/J (*Aldh1l1-CreER^T2^*, RRID: IMSR_JAX:029,655), B6.Cg-Tg(Gfap-cre)77.6Mvs/2J (*mGfap-Cre,* RRID: IMSR_JAX:024,098, Line77.6), *Sox2*^*tm1.1Lan*^/J (*Sox2*^*fl/fl*^, RRID: IMSR_JAX:013093), and B6.129X1-*Gt(ROSA)26Sor*^*tm1(EYFP)Cos*^/J (*Rosa26-EYFP, IMSR_JAX:006,148)* were described in our previous studies.^6,14,35,42^ The reporter line B6.129(Cg)-*Gt(ROSA)26Sor*^*tm4(ACTB-tdTomato,-EGFP)Luo*^/J (mTmG, RRID: IMSR_JAX:007,676) mice were purchased from JAX.^43^ Animal genotype was determined by PCR of genomic DNA extracted from tail tissue. All Cre lines were maintained as heterozygosity.

ALDH1L1 is a specific pan-astrocyte marker in the postnatal murine CNS.^44^ As such, tamoxifen-inducible Cre line, *Aldh1l1-CreER*^*T2*^, is the most widely used Cre driver that mediates gene recombination in postnatal astrocytes throughout the murine CNS. Our recent data of EYFP-based reporter line (*Aldh1l1-CreER*^*T2*^*:Rosa26-EYFP*) demonstrate that tamoxifen injection to postnatal mice elicits astrocyte-specific EYFP expression with 90% efficiency and nearly 100% specificity.^14^

In the *mGfap-Cre* line 77.6 mice^45^ (RRID: IMSR_JAX:024,098) used in our study, Cre recombinase activity, as defined by reporter genes, is targeted to most astrocytes throughout healthy brain and spinal cord tissues (www.jax.org/strain/024098), which is confirmed by our recent EYFP reporter fate-tracing data.^14^ In contrast to another widely used *mGfap-Cre* line 73.12 (RRID: IMSR_JAX:012886), *mGfap-Cre* line 77.6 mice are reported to have no Cre recombinase activity in postnatal or adult neural stem cells (or their progeny) from the hippocampus or other brain regions. Therefore, *mGfap-Cre* line 77.6 mice are particularly useful for selective targeting astrocytes.

In mTmG mice, a loxP-STOP-loxP sequence is placed before the coding frame of the membrane bound GFP (mGFP) reporter, preventing its expression. mGFP (mG) is expressed only in cells with nuclear Cre activity which excises the STOP code. In *Aldh1l1-CreER*^T2^:mTmG reporter mice, tamoxifen treatment drives Cre translocation into the nucleus of ALDH1L1^+^ astrocytes where Cre-mediated STOP deletion enables mGFP expression.

#### Generation of constitutive *Sox2* conditional knockout (Sox2 cKO) mice and inducible *Sox2* cKO (*Sox2* icKO) mice

##### Sox2 *cKO mice*

Female *mGfap-Cre* mice were bred with male *mGfap-Cre:Sox2*^*fl/fl*^ mice to generate double transgenic *mGfap-Cre:Sox2*^*fl/+*^ *mice.* Female *mGfap-Cre:Sox2*^*fl/+*^ mice were back-crossed with male *Sox2*^*fl/fl*^ mice to generate four types of transgenic mice: *mGfap-Cre: Sox2*^*fl/fl*^, *mGfap-Cre:Sox2*^*fl/+*^, *Sox2*^*fl/fl*^, and *Sox2*^*fl/+*^ mice with expected mendelian ratio (1:1:1:1). Our pilot study found no differences in astrocytic phenotypes and behaviors among *mGfap-Cre:Sox2*^*fl/+*^, *Sox2*^*fl/fl*^, and *Sox2*^*fl/+*^ mice. Therefore, mice carrying *mGfap-Cre:Sox2*^*fl/+*^, *Sox2*^*fl/fl*^ or *Sox2*^*fl/+*^ were pooled and used as control (Ctrl) mice, and those carrying *mGfap-Cre:Sox2*^*fl/fl*^ were used and designated as *mGfap:Sox2* cKO mice (referred to as *Sox2* cKO in the study).

##### Sox2 *icKO mice*

For tamoxifen-inducible *Sox2* icKO, we bred *Aldh1l1-CreER*^*T2*^ mice with *Sox2*^*fl/fl*^ mice to generate double transgenic *Aldh1l1-CreER*^*T2*^*:Sox2*^*fl/+*^ mice, which were back-crossed with *Sox2*^*fl/fl*^ mice to generate four types of transgenic mice: *Aldh1l1-CreER*^*T2*^*: Sox2*^*fl/fl*^*, Aldh1l1-CreER*^*T2*^*:Sox2*^*fl/+*^, *Sox2*^*fl/fl*^, and *Sox2*^*fl/+*^ mice. *Aldh1l1-CreER*^*T2*^*:Sox2*^*fl/fl*^ mice were used as *Aldh1l1:Sox2* icKO (referred to as *Sox2* icKO in the study) and those carrying *Aldh1l1-CreER*^*T2*^*:Sox2*^*fl/+*^, *Sox2*^*fl/fl*^, or *Sox2*^*fl/+*^ mice were pooled and used as control (Ctrl) mice. All study mice generated from the breeding paradigm of *Aldh1l1-CreER*^*T2*^ *and Sox2*^*fl/+*^ were received tamoxifen in the same doses and time points as indicated. The conclusions in the study were supported by *Sox2* cKO and *Sox2* icKO transgenic mice.

We used P13 mice for astrocyte proliferation and maturation, P19 mice for astrocyte morphological test, P30-35 mice for electrophysiological test, and P60 mice for behavior test.

#### Tamoxifen and EdU injection to Sox2 icKO and control (Ctrl) mice

Tamoxifen (TM) (Cat# T5648, Sigma) and 4-hydroxytamoxifen (OH-TM) (Cat# H7904, Sigma) were prepared in a mixture of ethanol and sunflower seed oil (1:9, v/v). The stock solution of TM and OH-TM were 30 mg/mL and 10 mg/mL, respectively. All study mice including Ctrl and *Sox2* icKO mice were received TM and OH-TM subcutaneously at a dose of 200 μg/g and 100 ug/g body weight, respectively. EdU (Cat# A10044, Thermo Fisher Scientific) was dissolved in 0.9% sterile saline at 10 mg/mL. To study the proliferation of astrocyte, EdU was intraperitoneally injected into *Sox2* cKO and Ctrl littermates (100 μg/g) from postnatal day 1 to day 3 (P1-3).

#### Isolation of primary astrocytes from mouse brain by magnetic-activated cell sorting (MACS)

##### Tissue dissociation

Primary astrocytes isolation and culture from mouse brain were performed according to protocols provided by Miltenyi Biotec (refer to www.miltenyibiotec.com) and published protocols.^46^ Single-cell suspensions from P7 mouse brain of Ctrl and *Sox2* cKO mice were prepared using Neural Tissue Dissociation Kit (P) (Cat# 130-092-628, Miltenyi Biotec, Germany) combined with the gentleMACS Dissociator (Cat# 130-093-235, Miltenyi Biotec, Germany) following manufacturers’ protocol. Mouse brain was collected and cut into 0.5 cm pieces using a scalpel and transferred into pre-heated gentleMACS C tube (Cat# 130-093-237, Miltenyi Biotec, Germany) containing 1950 uL of Enzyme mix 1. C tube was attached upside down onto the sleeve of the gentleMACS Dissociator. Brain tissue was dissociated according to the gentleMACS program, combined with three times of slow, continuous rotation at 37°C. After termination of the program, C Tube was detached from the gentleMACS Dissociator, and centrifuged briefly to collect the sample at the bottom of the tube. Digested tissues were resuspended and applied to a MACS SmartStrainer (70 μm) to remove cell clumps and obtain single-cell suspension. The MACS SmartStrainer was washed by additional 10 mL of HBSS (w) (HBSS with Ca^2+^ and Mg^2+^, Cat# 55021C, Sigma-Aldrich) to collect all the cells. Cell number was determined using Sceptor 3.0 Handheld Automated Cell counter (Cat# PHCC360KIT, Millipore) combined with Sceptor 3.0 Cell Counter Sensors (Cat# PHCC360050, Millipore).

Astrocytes were isolated from cell suspensions of mouse brain using the Anti-ACSA-2 (astrocyte cell surface antigen-2) MicroBeads (Cat# 130-097-678, Miltenyi Biotec, Germany) based on the expression of the ACSA-2 antigen.^47,48^ Cell suspension was centrifuged at 300 g for 10 min at room temperature, and supernatant was aspirated completely. Cell pellet was resuspended in 80 uL of 0.5% BSA/PBS buffer per 10^7^ total cells. Fc receptors were blocked using 10 uL of FcB Blocking Reagent per 10^7^ total cells for 10 min in the refrigerator at 4°C. 10 uL of Anti-ACSA-2 MicroBeads was then added into the cells, mixed well, and incubated for 15 min in the refrigerator at 4°C. Cells were washed by adding 1-2 mL of 0.5% BSA/PBS buffer per 10^7^ cells and centrifuged at 300 g for 10 min. Supernatant was aspirated completely. Cell pellet was then resuspended in 500 uL of 0.5% BSA/PBS buffer and proceeded to magnetic separation.

##### Magnetic separation

LS MACS column (max number of labeled cells 2x10^7^, max number of total cells 4x10^7^) was placed in the magnetic field of a MACS Separator (Cat#130-090-976, Miltenyi Biotec) and rinsed with 3 mL of 0.5% BSA/PBS buffer. Cell suspension was applied onto the column. Flow-through containing unlabeled cells was collected. The column was washed 3 times using 3 mL of 0.5% BSA/PBS buffer. Unlabeled cells were collected and combined with the flow-through, which is the ACSA-2-negative cell fraction. Column was removed from the separator and placed on a 15 mL tube. 5 mL of serum-free AstroMACS Medium (Cat# 130-117-031, Miltenyi Biotec, Germany) was added onto the column. Magnetically labeled cells was immediately flushed out by firmly pushing the plunger into the column, which is the ACSA-2-positive cell fraction. Astrocytes were then seeded into poly-L-lysine and laminin coated plates or glass slides at the concentration of 2x10^5^ cells/mL for culture. Culture medium was replaced 50% every other day. Astrocytes were cultured for 14 days *in vitro.*

Complete AstroMACS Medium (Cat# 130-117-031, Miltenyi Biotec, Germany) is an optimized serum-free primary mouse astrocytes medium, consisted of 50 mL MACS Neuro Medium, 1 mL MACS NeuroBrew-21 solution, 100 uL AstroMACS Supplement and 125 uL L-glutamine (200 mM), prepared according to the protocol. Cell culture dishes and glass slides were coated with 0.01% poly-L-lysine overnight at 37°C and washed three times with double-distilled water, and then coated with 10 ug/mL laminin overnight at 37°C and washed three times with double-distilled water, and dried under sterile conditions.

### METHOD DETAILS

#### Animal behavior assessment

Animals were habituated to the behavioral room for at least 30 min before the test began. Two months old *mGfap:Sox2* cKO (*Sox2* cKO) and Ctrl mice were firstly tested for motor skills on Rotarod and CatWalk, followed by locomotion and cognition test by open field and Barnes maze, respectively, and then spontaneous locomotion and social interaction test by HomeCage activity and three-chamber evaluations, respectively. Some test paradigms were also conducted on tamoxifen inducible *Aldh1l1:Sox2* cKO (*Sox2* icKO) and Ctrl mice at two months old which were administered with tamoxifen at P14-P16 (three injections, once a day) to induce astroglia-specific *Sox2* deletion.

##### Accelerating rotarod test

Accelerating rotarod test was used for assessing animal motor coordination and motor performance.^49,50^ The initial speed of rotarod was 4 rotations per minute (rpm) and the maximal speed was 40 rpm with 1.2 rpm incremental every 10 s. Mice were trained on rotating rod for two consecutive days (4 trials each day with 60 min interval between trials) followed by data collection on the day after 2-day training. The maximum duration of each trial was set to 300 s. The time on rod and maximal speed at which mice fell off the rod were recorded and averaged by 4 individual trials.

##### CatWalk gait analysis

The real-time video-tracked CatWalk XT system with automated data collection (Noldus Information Technology) was used to quantify animal waking gait. The camera gain was set to 20 and the detection threshold to 0.1. All runs through the walkway with duration between 0.50 and 5.00 s were considered as successful runs. The average of 3 successful runs was used for data plotting.

##### Open field locomotion test

Open field test was performed using a 42 × 42 × 37 cm Photobeam Activity System (PAS)-Open Field (San Diego instrument) which is equipped with a 16x16 PhotoBeam configuration and an automated data collection station. Animals were allowed for adaptation to the open field for 30 min prior to test. The test session was set to 30 min. The moving activity of animals in the open field during each session was real-time collected by PAS version 1.0. The PAS software package was used for calculating total travel distance and percent of active time (with 3-s cutoff) according to the manufacture instruction.

##### Barnes maze test

Spatial learning/memory and locomotion were examined by Barnes maze. Briefly, mouse was placed in the center of the maze (100 cm diameter) which containing twenty holes (10 cm diameter). The test was performed in a noise-free room with strong illumination (>300 LUX) and visual cues for the escaping hole leading to the escaping (goal) box. Animals were trained to locate the escaping box for five consecutive days (D1-D5) prior to formal testing on day 6 (D6). The animal activity was monitored by a camera controlled by the Ethovision tracking system (Ethovision XT.14, Noldus). Each animal was trained or tested for 2 trials per day with an interval of approximately 60 min. The maximal duration of each trial was 5 min unless the mice found the goal box. During the training days, mice were placed in the center of the maze and allowed for free exploration for 5 min after which they were gently guided to the goal box and kept there for 1 min. During the testing day, mice were allowed for exploration for 5 min and the escape box was relocated for 180°. The tracking program will be automatically terminated when the animals had all four paws inside the escaping box. Total errors before entering the escape box, latency to entering the goal box, total distance traveled (path length), and moving velocity on the maze were recorded and calculated using Ethovision XT.14 software.

##### Spontaneous HomeCage locomotion activity

The SmartCageTM system (L 29.8 x W 18.0 x H 12.8 cm) (AfaSci, Inc. Burlingame, CA, USA) was used for automated analysis of spontaneous activity in animal’s home cages. Animal was placed into the Home Cages and allowed to explore for 20 min. The home-cage activity variables (travel distance and active time) were determined by photobeam breaks and were analyzed automatically using CageScore software (AfaSci).

##### Three-chamber social interaction test

Social interaction was assessed by using the SmartCageTM system with two add-on metal-mesh enclosure (W 8 x L 6 x H 12 cm) at each end of the Home Cage (see Figure S2D1), which has been shown equivalent to conventional “three-chamber” test in evaluating animal social interaction. The social interaction test consists of three 10-min sessions (habituation, sociability, social novelty, or memory) (see Figure S2D1). During habituation, the subject mouse was allowed to move around for 10 min without social cues. During sociability test, Stranger 1 mouse was placed into one of the two enclosures. The subject mouse was then allowed to explore for 10 min. The social novelty and memory were tested by introducing the unfamiliar Stranger 2 mouse and familiar Stranger 1 mouse after 1 h interval. The percentage of occupancy time spend with empty or stranger mouse were recorded by the SmartCage System (AfaSci, Inc. Burlingame, CA, USA).

#### RNA-sequencing (RNA-seq) and bioinformatic analysis

Total RNA was prepared from the spinal cord of P80 *mGfap:Sox2*^*fl/fl*^ (*Sox2* cKO, n = 3, 2 females and 1 male) and *Sox2*^*fl/fl*^ littermate control (Ctrl) mice (n = 3, 2 females and 1 male). The quality of RNA samples was determined by the Bioanalyzer 2100 system (Agilent Technologies). The RIN (RNA integrity number) of all our RNA samples used for RNA-seq and qPCR was greater than 8.0. The library constructing and sequencing, and subsequent bioinformatic analysis were conducted using the standard operating procedure by Novogene Inc. In brief, the library was prepared using the NEBNext Ultra Directional RNA Library Prep Kit (#E7420) for Illumina and sequenced on the Illumina HiSeq 4000 sequencing platform. Single-end clean reads were aligned to the mouse genome (mm10) using the STAR software package (v2.5) with mismatch = 2. HTSeq v0.6.1 was used to count the read numbers mapped of each gene and FPKM of each gene was calculated based on the length of the gene and reads count mapped to that gene. Differential expression analysis between *Sox2* cKO and Ctrl mice was performed using the DESeq2 R package (2.1.6.3), and genes with an adjusted p-value <0.05 found by DESeq2 were assigned as differentially expressed genes (DEGs). Gene Ontology (GO) enrichment analysis of DEGs was implemented by the clusterProfiler R package (v2.34.3), in which gene length bias was adjusted. GO terms with adjusted p value less than 0.05 were considered significantly enriched. The RNA sequencing data from this study have been deposited to GEO with the identification GSE182032.

#### Astrocyte morphology analysis

The data of Figures 2B-2H were quantified by IMARIS (Bitplane version 9.30). Briefly, z stack images were obtained by Nikon C2 confocal using 60x oil-immersion objective lens. The parameter setting of z stack confocal imaging is below: total optical thickness, 10 μm, step size, 0.5 μm, total number optical slices, 21. The original z stack images from Nikon C2 were imported to IMARIS for 3D morphological reconstruction according to the software manual. We manually adjusted the sensitivity threshold to obtain 3D images matched with original mGFP^+^ astrocytes from confocal images. To quantify astrocyte surface area and enclosed volume (domain), the Surface Tool of IMARIS was used to build the domains of mGFP^+^ astrocytes. Astrocytic cell soma was identified by S100β immunoreactive signals and automatically selected based on the size point (<12 μm). The Classification/Filter function of IMARIS was used to remove the background noise.

To quantify astrocyte processes, the IMARIS Filament Tracing module was performed to detect an automatic intensity threshold, subtract background noise, and generate process of astrocyte computer reconstructions which matched with original images. IMARIS parameters were set to detect processes between 0.6 μm and 10.4 μm in diameter. The process length, area, and volume of mGFP^+^ astrocytes were calculated automatically by IMARIS with same intensity threshold between *Sox2* icKO and Ctrl mice.

The data of Figure S3A4 were quantified by NIH ImageJ. Ten-micron optical sections from confocal z stack (Nikon C1) were projected into a flattened image. The parameter setting of z stack confocal imaging was below: total optical thickness, 10 μm, step size, 0.5 μm, total number optical slices, 21. The volume-rendered confocal images were subsequently imported to NIH ImageJ 1.46r for quantifying the percentage of astrocyte marker occupying area among total area. We used a customer-defined ImageJ Macro program according to our previous protocol for semi-automated quantification (Zhang et al., 2020). At least three sections from each mouse were used for ImageJ quantification.

#### Bioinformatic analysis of SOX2 ChIP-seq data

The dataset GSE85213,^8^ which consists of raw sequence reads of one input sample and 3 biological replicate samples of SOX2 IP prepared from the adult cerebral cortex, was used for bioinformatics analysis. ChIP-seq raw reads of H3K27Ac in cultured mature astrocytes at DIV21 differentiation from mouse embryonic stem cells were retrieved from the dataset GSE96539 (Tiwari et al., 2018). We used the open source, web-based platform usegalaxy.org for ChIP-seq data analysis.

For SOX2 ChIP-seq analysis, bowtie 2 was used to align raw reads to the mouse reference genome mm10 and the resulting aligned reads were written into BAM files, which were subsequently filtered by minimal MAPQ quality score >20 (filtered BAM files). Bigwig files were generated based on filtered BAM files using bamCoverage tool. MACS2 was used for peak calling with the following parameters: ChIP-seq Treatment File (IP-1, IP-2, and IP-3); ChIP-seq Control File (Input); effective genome size, 2,150,570,000; bandwidth for picking regions to compute fragment size, 300; lower mfold bound, 5; upper mfold bound, 100; peak detection based on q-value (FDR) with minimal q-value cutoff for peak detection 0.05. The resulting NarrowPeak BED files were used for subsequent bioinformatic analysis. The gene annotation of the identified peaks to the closest transcription start sites (TSS) was conducted using the online tool annoPeakR (Tables S1 and S2). We used MEME-CHIP (http://meme-suite.org/tools/meme-chip) for motif analysis (Table S3) and GREAT v3.0 (http://great.stanford.edu/great/public-3.0.0/html/) for biological function annotation (Table S4).

#### Tissue collection

Mice were anesthetized by ketamine/xylazine mixture and transcardially perfused with ice-cold PBS. Harvested brain and spinal cord were placed immediately on dry ice for protein or RNA extraction or fixed in fresh 4% paraformaldehyde (PFA) for histological study. After fixation in 4% PFA overnight at 4°C, tissues were washed with PBS three times, 15 min per time and cryopreserved in 30% sucrose in PBS overnight at 4°C followed by embedding in O.C.T. (Cat# 361603E, VWR International). Serial coronal sections (12 μm) were cut using a Leica Cryostat (CM1900-3-1). All slides were stored in the −80°C refrigerator.

#### Immunofluorescence (IF) staining

Tissue slides were air-dried for 2 h and blocked in 8% donkey serum in 0.1% Triton X-100/PBS (v/v) for 1 h at room temperature followed by primary antibody incubation at 4°C overnight. After three times of washing with 0.1% Tween 20/PBS (v/v) (PBST), slices were incubated with secondary antibodies for 2 h at room temperature. DAPI was used as a nuclear counterstain. Images were taken using a Nikon A1 confocal microscope. A z stack of optical sections, 10 μm in total thickness, were collapsed into a single 2D image for quantification. The following antibodies were used for our IHC study: SOX2 (1:500, Santa Cruz biotechnology); BLBP (1:200, Millpore); GFAP (1:500, Millpore); GFAP (1:500, Agilent); SOX9 (1:200, R&D system); Sox10 (1:300, Abcam); EYFP/GFP (1:500, abcam); Kir4.1 (1:400, Alomone labs); Sox9 (1:500, R&D system); VGlut1 (1:500, Millipore); PSD95 (1:500, Synaptic Systems); NeuN (1:500, Millipore); c-Fos (1:500, Santa Cruz Biotechnology). All secondary antibodies Alexa Fluor 488- or Alexa Fluor 594-conjugated AffiniPure F(ab’)2 fragments (1:500) were from Jackson ImmunoResarch Laboratories.

#### Histochemistry

For the visualization of GLT-1, a 3,3′-diaminobenzidine (DAB) Substrate Kit (Cat# SK-4100, Vector Labs) was performed. Endogenous peroxidases were inactivated by treating slices with 1% H_2_O_2_ in methanol for 5 min. After blocking with 10% normal donkey serum containing 0.1% Triton X-100 for 1 h, the slices were incubated overnight with primary antibody at 4°C. Slides were washed three times for 10 min each time wash PBST, incubated with appropriate secondary biotinylated antibody (Vector Labs), and visualized using DAB substrates. Finally, slides were rinsed with PBST to remove extra DAB mixture and carefully dehydrated using ethanol in the following sequence: 70% ethanol, 15 min; 80% ethanol, 15 min; 90% ethanol 15 min; 100% Ethanol for twice, each time for 10 min; followed by xylene for 2 times. The slides were mounted with Permount Mounting medium (Cat# SP15-100, Fisher Scientific).

#### Immunocytochemistry (ICC) staining

Primary astrocytes cultured on glass slides were fixed in 4% PFA for 30 min, permeabilized with 0.1% Triton X-100 in PBS and blocked with 10% donkey serum for 1 h at room temperature. The cells were then washed with PBS and incubated with primary antibodies overnight at 4°C, followed by fluorescence-conjugated secondary antibodies (1:200) incubation for 2 h at room temperature. Nuclei were visualized using DAPI. Fluorescent images were observed with Nikon A1 confocal microscope.

#### *In situ* hybridization

A 614 bp digoxin-conjugated complementary RNA (cRNA) probe (targeting *Gfap* mRNA) was prepared using SP6 RNA polymerase-mediated *in vitro* transcription. The DNA template for *in vitro* transcription was prepared using *Gfap* mRNA-specific forward primers 5′-GTGGATTTGGAGAGAAAGGTTG-3′ and reverse primer 5′- GCGATTTAGGTGACACTATAGCTGGAGGTTGGAGAAAGTCTGT-3′ (underlined is the core sequence recognized by SP6 RNA polymerase). The *in vitro* transcription was performed and used as templates according to the instructions of DIG RNA labeling Kit (SP6/T7, Cat# 11175025910, Roche). Frozen sections (14 μm thickness) were used for mRNA ISH. The hybridization was performed at 65°C for 12 to 18 h. The sections were washed by washing solution containing saline sodium citrate buffer (SSC) three times 10 min per time and immersed in RNases solution at 37°C for 30 min to eliminate non-specific binding and non-hybridized mRNAs. The sections were then incubated in NBT/BCIP solution (Cat# 11681451001, Roche) according to the manufacturer’s instruction at 4°C overnight followed by blocking with blocking buffer for 1 h at room temperature and incubated with anti-Digoxigenin Fab fragments antibody (Cat#11093274910, Roche) at 4°C overnight. The DIG-conjugated mRNA signals were visualized by HNPP/Fast Red Fluorescent Detection set (Cat# 11758888001, Roche) according to the kit instructions.

#### Protein extraction and Western blot

Frozen tissues were lysed in N-PER Neuronal Protein Extraction Reagent (Thermo Fisher) containing protease and phosphatase inhibitor cocktail (Cat# PPC1010, Thermo Fisher) and PMSF (Cat# 8553, Cell Signaling Technology) on ice for 10 min and centrifuged at 13,000 g for 10 min at 4°C to pellet the insoluble material and cell debris. Supernatants were collected and protein concentrations were measured using BCA protein assay Kit (Cat# 23225, ThermoFisher Scientific). Equal amounts of protein (30 μg) from each sample were loaded into SDS-PAGE gels (BIO-RAD) for electrophoresis. The proteins were transferred onto a 0.2 μm nitrocellulose membrane (Cat# 1704158, BIO-RAD) using Trans-blot Turbo Transfer system (Cat# 1704150, Bio-Rad). The membrane was then blocked in 5% BSA for 1 h at room temperature, and incubated with primary antibodies overnight at 4°C. After three times of 10 min wash in PBST, the membrane was incubated in horseradish peroxidase (HRP)-conjugated secondary antibodies. The membrane was imaged using Western Lightening Plus ECL (Cat# NEL 103001EA, PerkinElmer). The intensities of the bands were quantified by ImageJ software to analyzing the scanned grayscale value. Antibodies used were: β-actin (1:1000, Cell Signaling Technology), GAPDH (1:2000, Cell Signaling Technology), GFAP (1:1000, Millpore), SPARCL1 (1:2000, R&D system), Kir4.1 (1:1000, Alomone labs), GLT-1 (1:5000, rabbit) antibody was generously provided by J.D. Rothstein (John Hopkins University, Baltimore, MD). Multiple GLT-1 protein bands of ~62 kD, ~120 kDa, and ~250 kDa were observed in Western blot images, representing monomer, dimer, and tetramer of GLT-1 molecules as described in previous study.^51^

#### RNA isolation, cDNA preparation, and real-time quantitative PCR (RT-qPCR)

Tissues were added with 1 mL of QiaZol (Cat# 79306, QIAGEN) and homogenized using BEADBUG 6 microtube homogenizer (Benchmark Scientific, USA). Total RNA was isolated with RNeasy Lipid Tissue Mini Kit (Cat#74804, QIAGEN) following the manufacturer’s protocol. An efficient on-column DNase treatment during RNA purification with RNase-Free DNase Set (Cat#79254, QIAGEN) was included to eliminate potential DNA contamination. 35 μL of RNase-free water was added to the spin column membrane and centrifuged for 2 min at 14,000 rpm to elute the RNA. The RNA concentration and purity (260/280 and 260/230 ratios) were analyzed using a Nanodrop 2000 Spectrophotometer (Thermo Fisher Scientific). cDNA was synthesized from 2000 ng of total RNA using QIAGEN Omniscript RT Kit (Cat#205111, QIAGEN) according to the manufacturer’s guidelines. The cDNA amplification was performed with a QuantiTect SYBR Green PCR Kit (Cat#204145, QIAGEN). The real-time PCR reaction was performed and analyzed by using Agilent MP3005P thermocycler. Primer sets (Please see Table S7) were designed according to PrimerBank sequences and were ordered from Integrated DNA Technologies. For quantification, the mRNA expression level of target genes in each sample was normalized to that of the *Hsp90* as a reference gene, and the fold change of gene expression level was calculated based on the Equation 2^(Ct(cycle threshold) of Hsp90-Ct of indicated genes.

#### Chromatin immunoprecipitation (ChIP) assay

ChIP-qPCR assay was performed using SimpleChIP Enzymatic Chromatin IP Kit (Cat# 9003; Cell Signaling Technologies) according to manufacturer’s protocol. Primary astrocyte samples were crosslinked with 37% formaldehyde at a final concentration of 1% for 10 min at room temperature and subsequently quenched with 125 mM glycine for 5 min. The cells were centrifuged and washed twice with ice-cold PBS before being harvested in PBS with protease inhibitor cocktail and lysed to get nuclei. Nuclei were extracted and incubated with Micrococcal Nuclease to digest the Chromatin to fragments of between an optimal 150 and 900 bp length. The nuclei were sonicated using the Sonic equipment to break down the nuclear membrane. The digested chromatin was collected and then subjected to IP with SOX2 antibody or normal goat IgG (Cat# AB-108-C, R&D Systems) as control overnight at 4°C with rotation. Protein G magnetic bead were added to each sample and incubated for 2 h at 4°C with rotation. After washing, chromatin was eluted from primary antibody/protein G magnetic beads at 65°C for 30 min with vortexing in a thermomixer (Thermo Fisher Scientific). The protein-DNA cross-links were reversed by incubating with proteinase K overnight at 65°C to remove the proteins. The reverse cross-linking DNA was purified and subjected by RT-qPCR. The primers used for ChIP-qPCR were designed according to the ChIP-sequencing results and listed in Table S7.

#### Astrocyte glutamate uptake

Glutamate uptake was performed using DIV14 primary astrocytes cultured in serum-free medium. Cells were washed with HBSS (with Ca^2+^ and D-glucose) and equilibrated with HBSS for 15 min at 37°C in the 5% CO_2_ incubator. Cells were treated with 100 μM glutamate (Cat# PHR1007, Sigma) freshly dissolved in HBSS for 1.5 or 4 h. Glutamate concentration was determined by using the commercial glutamate assay Kit (Cat# MAK004, Sigma) according to the manufacture instructions. Briefly, cells were washed with cold HBSS three times, homogenized in 100 μL of the Glutamate Assay Buffer, and centrifuged at 13,000 g for 10 min to remove insoluble material. 50 uL of cell lysates were used for glutamate uptake assay, and the rest of cell lysates were used for whole cell protein concentration assay by BCA method. The glutamate uptake was normalized to cell protein concentration and calculated as nmol/mg total protein.

#### Synapse quantification at the histological levels

Frozen sections (12 μm) were immune-stained for the presynaptic marker VGlut1 and postsynaptic marker PSD95 and imaged by Nikon C1 confocal with the following z stack settings: optical thickness of 2 μm and z-step size of 0.2 μm. The maximally projected images from 5 consecutive optical slices (total depth, 0.2 μm x 5 optical slices = 1 μm optical thickness) were analyzed by Puncta Analyzer plug-in of NIH ImageJ. We used the protocol previously published by Dr. Eroglu (Duke University)^52^ to quantify the synaptic puncta density. As described in the published protocol,^52^ we normalized the synapse puncta density in control mice to 100%.

#### Electrophysiology recording of astrocytes and neurons

Mice of 4-5 postnatal weeks were used for electrophysiological recordings. After anesthesia with Ketamine/Xylazine combination (100-200 mg/kg body weight for Ketamine and 5-16 mg/kg body weight for Xylazine injected intraperitoneally), mice were decapitated and the brain was rapidly collected and placed into ice-cold slicing solution (125 mM NaCl, 3.5 mM KCl, 25 mM NaHCO_3_, 1.25 mM NaH_2_PO_4_, 0.1 mM CaCl_2_, 3 mM MgCl_2_, and 10 mM Glucose) oxygenated with 95% O_2_ and 5% CO_2_. The oxygenated brain was mounted on the Vibratome (Cat# VT100S, Leica). Sagittal brain slices (300 μm thickness) were sectioned in the ice-cold slicing solution and then transferred into a small basket in a container within oxygenated standard artificial cerebrospinal fluid (aCSF, 125 nM NaCl, 25 nM NaHCO_3_, 1.25 nM NaH_2_PO_4_, 3.5 nM KCl, 2 mM CaCl_2_, 1 mM MgCl_2_, and 10 nM Glucose, 295 ± 5 mΩ; pH 7.3-7.4; oxygenated with 95% O_2_ and 5% CO_2_), incubated at 32°C for 30 min and then recovered from preparation damage at room temperature for 1 h. To visualize astrocytes, brain slices were briefly incubated in the oxygenated standard aCSF supplemented with 0.6μM astrocytic marker sulforhodamine 101 (Cat# S7635, Sigma-Aldrich) for 30 min.

Whole-cell patch-clamp recordings of cortical astrocyte and dorsal striatal medium spiny neurons (MSNs) were conducted using MultiClamp 700B amplifier, filtered at 3 kHz, and sampled at 10 kHz with DigiData 1440 (Molecular Devices). Brain slices were continuously perfused with aCSF with a flow rate of 2-3 mL/min bubbled with 95% O_2_ and 5% CO_2_. For cortical astrocyte recording, the glass pipette resistance was 5-6 mΩ. Input resistance and capacitance were measured in voltage-clamp mode with 500 ms 5 mV step pulse from −70 mV holding potential. The resting membrane potential was measured in current-clamp model (I = 0). For neuron recording, MSNs were identified by bright-field Nikon Eclipse e600FN microscopy with a 40 water-immersion lens (numerical aperture 0.8) and infrared illumination. To measure the miniature excitatory postsynaptic current (mEPSCs), the glass pipette resistance was 3-5 mΩ, and MSNs were recorded in voltage-clamp mode at a holding potential of −70 mV in the presence of 1 μM (Tetrodoxin) TTX and 50 μM picrotoxin (GABA antagonist). pClamp 11.0.3 (Molecular Devices) was used for data acquisition and storage. Analysis of mEPSCs was performed using Minianalysis 6.0.7 software (Synaptosoft). At least 5 min of spontaneous activity was recorded from each cell with access resistance measured before and after the recording. Access resistance (Ra) was monitored following membrane rupture and dialysis, and recordings were abandoned if Ra >30 M.

### QUANTIFICATION AND STATISTICAL ANALYSIS

Both male and female mice were included in this study. All data were plotted as mean ± s.e.m. Data collection was conducted by lab members who were blinded to mouse genotypes. We used Shapiro-Wilk approach to test data normality. F test and Browne-Forsythe test were used to test variance equality of two groups and three or more groups, respectively. Unpaired two-tailed Student’s *t* test was used for statistically analyzing two groups of data where the *t*-values and the degree of freedom (df) were shown as t_(df)_ in each graph. two-way ANOVA followed by Tukey’s post-test was used for statistically analyzing the data of Figure S2A2-3 and S2D2-3 where the F ratio, and DFn and DFd was presented as F(DFn, DFd) in the figure legends. All data graphing and statistical analyses were performed using GraphPad Prism version 8.0. The p-value was designated as *p < 0.05, **p < 0.01, ***p < 0.001, ns, not significant p > 0.05.

## Supplementary Material

1

2

4

5

6

7

8

9

10

11

## Figures and Tables

**Table:** KEY RESOURCES TABLE

REAGENT or RESOURCE	SOURCE	IDENTIFIER
Antibodies		
Goat polyclonal SOX2 (Y-17)	Santa Cruz Biotechnology	Cat# sc-17320; RRID: AB_2286684
Rabbit polyclonal anti-BLBP	Millipore	Cat#ABN14; RRID: AB_2494022
Mouse monoclonal anti-GFAP	Millipore	Cat# MAB36014; RRID: AB_11212597
Rabbit polyclonal anti-GFAP	Agilent	Cat# Z0334; RRID: AB_10013382
Rabbit polyclonal SOX10	Abcam	Cat# ab27655; RRID: AB_778021
Goat polyclonal anti-SOX9	R&D system	Cat# AF3075; RRID: AB_2194160
Mouse polyclonal anti-SPARCL1	R&D system	Cat# AF2836; RRID: AB_2195097
Chicken polyclonal anti-GFP	Abcam	Cat# ab13970; RRID: AB_300798
Rabbit polyclonal anti-Kir4.1	Alomone labs	Cat# APC-035; RRID: AB_2040120
Rabbit polyclonal anti-GLT1	Provided by J.D. Rothstein	Higashimori et al., 2016
Guinea pig polyclonal anti-VGLUT1	Millipore	Cat# AB5905; RRID: AB_2301751
Rabbit monoclonal anti-S100beta	Abcam	Cat#ab52642 RRID: AB_882426
Mouse monoclonal anti-PSD95	Synaptic Systems	Cat# 124011; RRID: AB_10804286
Mouse monoclonal anti-NeuN	Millipore	Cat# MAB377; RRID: AB_2298772
Rabbit polyclonal c-Fos	Santa Cruz Biotechnology	Cat# sc-52; RRID: AB_2106783
Rabbit polyclonal Anti-GLAST	Proteintech	Cat# 20785-1-AP; RRID: AB_2878738
Mouse monoclonal β-actin	Cell Signaling Technology	Cat# 3700; RRID: AB_2242334
Rabbit monoclonal anti-GAPDH	Cell Signaling Technology	Cat# 2118; RRID: AB_561053
HRP goat anti-rabbit IgG (H + L)	Thermo Fisher Scientific	Cat# 31460; RRID: AB_228341
HRP goat anti-mouse IgG (H + L)	Thermo Fisher Scientific	Cat# 31430; RRID: AB_228307
Normal goat IgG control	R&D Systems	Cat# AB-108-C; RRID: AB_354267
Alexa Fluor^®^ 488 AffiniPure F(ab’)_2_ Fragment Donkey Anti-Goat IgG (H + L)	Jackson ImmunoResearch Laboratories	Cat#705-546-147; RRID: AB_2340430
Alexa Fluor^®^ 594 AffiniPure F(ab’)_2_ Fragment Donkey Anti-Goat IgG (H + L)	Jackson ImmunoResearch Laboratories	Cat# 705-586-147; RRID: AB_2340434
Alexa Fluor^®^ 488 AffiniPure F(ab’)_2_ Fragment Donkey Anti-Rabbit IgG (H + L)	Jackson ImmunoResearch Laboratories	Cat# 711-546-152; RRID: AB_2340619
Alexa Fluor^®^ 488 AffiniPure F(ab’)_2_ Fragment Donkey Anti-Mouse IgG (H + L)	Jackson ImmunoResearch Laboratories	Cat# 715-546-150; RRID: AB_2340849
Alexa Fluor^®^ 594 AffiniPure F(ab’)_2_ Fragment Donkey Anti-Mouse IgG (H + L)	Jackson ImmunoResearch Laboratories	Cat# 715-586-150; RRID: AB_2340857
Biotin-SP (long spacer) AffiniPure F(ab’)_2_ Fragment Donkey Anti-Goat IgG (H + L)	Jackson ImmunoResearch Laboratories	Cat# 705-066-147; RRID: AB_2340398
Alexa Fluor^®^ 594 AffiniPure F(ab’)_2_ Fragment Donkey Anti-Guinea pig IgG (H + L)	Jackson ImmunoResearch Laboratories	Cat# 706-586-148; RRID: AB_2340475
Alexa Fluor^®^ 488 AffiniPure F(ab’)_2_ Fragment Donkey Anti-Chicken IgG (H + L)	Jackson ImmunoResearch Laboratories	Cat# 703-546-155; RRID: AB_2340376
Sheep Anti-Digoxigenin Fab fragments Antibody, AP Conjugated	Roche	Cat# 11093274910; RRID: AB_514497
N-PER^™^ Neuronal Protein Extraction Reagent	Thermo Fisher	Cat# 87792
Chemicals, peptides, and recombinant proteins		
Tamoxifen	Sigma-Aldrich	Cat# T5648
4-hydroxytamoxifen	Sigma-Aldrich	Cat# H7904
Sunflower seed oil	Sigma-Aldrich	Cat# 47123
5-Ethynyl-2′-deoxyuridine (EDU)	Thermo Fisher Scientific	Cat# A10044
10x PBS	K-D Medical	Cat# RGF-3210
Paraformaldehyde	Electron Microscopy Science	Cat# 1570-S
O.C.T. compound	VWR International	Cat# 361603E
Normal donkey serum	Jackson ImmunoResearch Laboratories	Cat# 017-000-121
DAPI	Sigma-Aldrich	Cat# D9542
Xylene	Sigma-Aldrich	Cat# 534056
Mounting medium	Thermo Fisher Scientific	Cat# SP15-100
Poly-L-lysine	Sigma-Aldrich	Cat# P4707
laminin	Sigma-Aldrich	Cat# L2020
High-glucose DMEM medium	Gibco	Cat# 11965-092
BSA	Cell Signaling Technology	Cat# 9998
Penicillin/Streptomycin	Thermo Fisher Scientific	Cat# 15140122
Trypsin-EDTA	Promega	Cat# V528A
Fetal bovine serum	Sigma-Aldrich	Cat# 12306-C
HBSS	Sigma-Aldrich	Cat# 55021C
Ketamine	VetOne	Cat# 13985-584-10
Xylazine	AnaSed Injection	Cat# 59399-110-20
Sulforhodamine 101	Sigma-Aldrich	Cat# S7635
Tarichatoxin	Sigma-Aldrich	Cat# T8024
Picrotoxin	Sigma-Aldrich	Cat# P1675
Protease and phosphatase inhibitor cocktail	Thermo Fisher	Cat# PPC1010
10X Tris/Glycine/SDS buffer	BIO-RAD	Cat# 1610772
Tween 20	Sigma-Aldrich	Cat# T8787
Triton X-100	Sigma-Aldrich	Cat# P9416
AstroMACS Medium	Miltenyi Biotec	Cat# 130-117-031
MACS^®^ Neuro Medium	Miltenyi Biotec	Cat# 130-093-570
L-Glutamine Solution 200 mM	Sigma-Aldrich	Cat# 56-85-9
NBT/BCIP stocking solution	Roche	Cat# 11681451001
PMSF	Cell Signaling Technology	Cat# 8553
QIAzol Lysis Reagent	QIAGEN	Cat# 79306
Glutamate	Sigma	Cat# PHR1007
Streptavidin, Pacific Blue^™^ conjugate	Thermo Fisher Scientific	Cat# S11222
Critical commercial assays		
BCA protein assay Kit	Thermo Fisher Scientific	Cat# 23225
Western Lightening Plus ECL	Perkin Elmer	Cat# NEL103001EA
Commercial glutamate assay Kit	Sigma-Aldrich	Cat# MAK004
NEBNext Ultra Directional RNA Library Prep Kit	New England BioLabs Inc	Cat# E7420
DIG RNA labeling Kit	Roche	Cat# 11175025910
RNeasy Lipid Tissue Mini Kit	Qiagen	Cat# 74804
RNase-Free DNase Set	Qiagen	Cat# 79254
Qiagen Omniscript RT Kit	Qiagen	Cat# 205111
QuantiTect SYBR^®^ Green PCR Kit	Qiagen	Cat# 204145
SimpleChIP Enzymatic Chromatin IP Kit	Cell Signaling Technologies	Cat# 9003
HNPP/Fast Red Fluorescent Detection set	Roche	Cat# 11758888001
Neural Tissue Dissociation Kit (P)	Miltenyi Biotec	Cat# 130-092-628
Anti-ACSA-2 MicroBead Kit	Miltenyi Biotec	Cat# 130-097-678
Trans-blot turbo RTA midi 0.2 μm nitrocellulose transfer Kit	BIO-RAD	Cat# 1704271
Deposited data		
RNA-seq	Guo Lab	Datebase: GSE182032
ChIP-seq	Zhang Lab	Datebase: GSE85213
ChIP-seq	Berninger Lab	Datebase: GSE96539
Experimental models: Organisms/strains		
B6; FVB-Tg(Aldh1l1-cre/ERT2)1Khakh/J	Jackson Laboratory	RRID: IMSR_JAX:029,655
B6.Cg-Tg(Gfap-cre)77.6Mvs/2J	Jackson Laboratory	RRID: IMSR_JAX:024098,
*Sox2^tm1.1Lan^*/J	Jackson Laboratory	RRID: IMSR_JAX:013,093
B6.129X1-*Gt(ROSA)26Sor^tm1(EYFP)Cos^*/J	Jackson Laboratory	RRID: IMSR_JAX:006,148
B6.129(Cg)-*Gt(ROSA)26Sor^tm4(ACTB-tdTomato,-EGFP)Luo^*/J	Jackson Laboratory	RRID: IMSR_JAX:007,676
Oligonucleotides		
See Table S7		
Software and algorithms		
ImageJ	ImageJ public	https://imagej.net/Fiji/Downloads
IMARIS version 9.30	Oxford instruments	https://imaris.oxinst.com/newrelease
MEME-CHIP Version 5.4.1	Noble research lab	http://meme-suite.org/tools/meme-chip
GREAT Version 3.0	Bejerano Lab	http://great.stanford.edu/great/public-3.0.0/html/
DESeq2 R package vesion 2.1.6.3	Bioconductor	https://bioconductor.org/packages/release/bioc/html/DESeq2.html
ClusterProfiler R package version 2.34.3	Bioconductor	https://bioconductor.org/packages/release/bioc/html/clusterProfiler.html
CatWalk XT	Noldus Information Technology	https://www.noldus.com/catwalk-xt
Photobeam Activity System	San Diego instrument	https://sandiegoinstruments.com/product/pas-open-field/
Ethovision tracking software system vesion XT.14	Noldus	https://www.noldus.com/ethovision-xt
SmartCageTM system	AfaSci, Inc.	https://www.afasci.com/index.php/instruments/smartcage
Minianalysis 6.0.7 software	Synaptosoft	http://www.synaptosoft.com/MiniAnalysis/
GraphPad Prism version 8.0	GraphPad Software	https://www.graphpad.com/company
Bioanalyzer 2100 system	Agilent Technologies	https://www.agilent.com

## Highlights

- Astroglial *Sox2* deletion leads to hyperactivity in locomotion
- Astroglial *Sox2* deletion perturbs postnatal astrocyte maturation
- SOX2 regulates the expression of a cohort of astrocytic signature and functional genes
- Astroglial *Sox2* deletion compromises astroglial glutamate uptake
