# Supplementary material for: SOX2 is essential for astrocyte maturation and its deletion leads to hyperactive behavior in mice
Source: Cell Rep. Author manuscript; Available in PMC 2023 Jan 25. (PMC9875714; doi:10.1016/j.celrep.2022.111842)
Supplement: 1 [file NIHMS1860001-supplement-1.pdf]

Cell Reports, Volume *41*

Supplemental information

SOX2 is essential for astrocyte maturation and its deletion leads to hyperactive behavior in mice

Yan Wang, Sheng Zhang, Zhaohui Lan, Vui Doan, Bokyung Kim, Sihan Liu, Meina Zhu, Vanessa L. Hull, Sami Rihani, Chun-Li Zhang, John A. Gray, and Fuzheng Guo

Supplemental Figures and Legends

Supplemental Figures and Legends

Figure S1 – Efficiency and specificity of *Sox2* deletion in *Sox2* cKO and *Sox2* icKO mice (related to Figure 1-4)

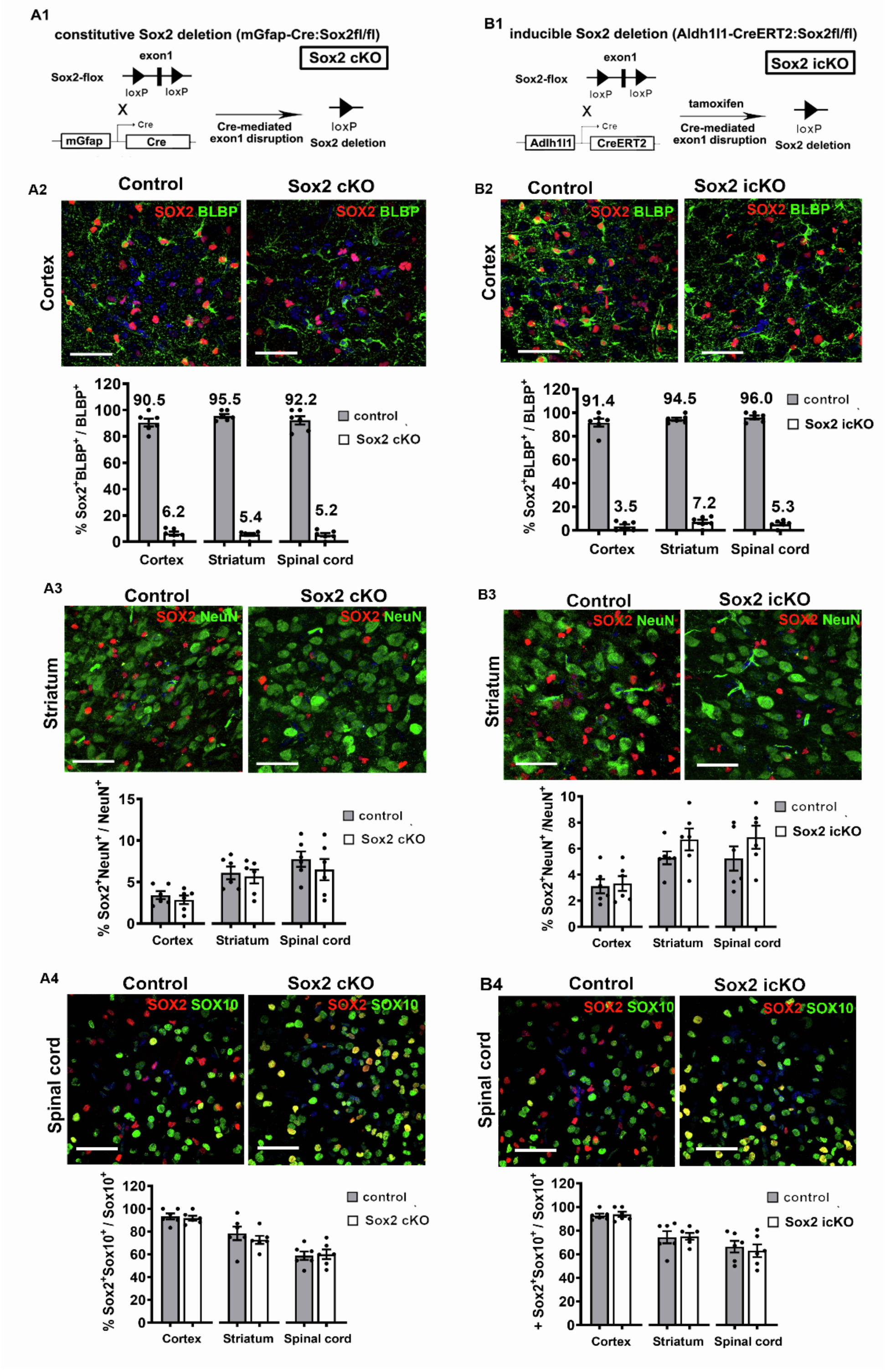

Supplemental Figures and Legends

(A1) Diagram of constitutive *Sox2* cKO in astrocytes of *mGfap-Cre Sox2*$^{fl/fl}$ mice.
(A2) Immunofluorescence of SOX2/BLBP in the cerebral cortex of control and *Sox2* cKO mice. Quantification of SOX2^{+}/BLBP^{+} cells within the total BLBP^{+} population in control and *Sox2* cKO cortex, striatum, and spinal cord.
(A3) Immunofluorescence of SOX2/NeuN in the striatum of control and *Sox2* cKO mice. Quantification of SOX2^{+}/NeuN^{+} cells within the total NeuN^{+} population in control and *Sox2* cKO cortex, striatum, and spinal cord.
(A4) Immunofluorescence of SOX2/SOX10 in the striatum of control and *Sox2* cKO mice. Quantification of SOX2^{+}/SOX10^{+} cells within the total SOX10^{+} population in control and *Sox2* cKO cortex, striatum, and spinal cord.
(B1) Diagram of inducible *Sox2* cKO in astrocytes of *Aldh1l1-CreERT2 Sox2*$^{fl/fl}$ mice.
(B2) Immunofluorescence of SOX2/BLBP in the cerebral cortex of control and *Sox2* icKO mice. Quantification of SOX2^{+}/BLBP^{+} cells within the total BLBP^{+} population in control and *Sox2* icKO cortex, striatum, and spinal cord.
(B3) Immunofluorescence of SOX2/NeuN in the striatum of control and *Sox2* icKO mice. Quantification of SOX2^{+}/NeuN^{+} cells within the total NeuN^{+} population in control and *Sox2* icKO cortex, striatum, and spinal cord.
(B4) Immunofluorescence of SOX2/SOX10 in the striatum of control and *Sox2* icKO mice. Quantification of SOX2^{+}/SOX10^{+} cells within the total SOX10^{+} population in control and *Sox2* icKO cortex, striatum, and spinal cord.
Scale bar, 50 μm.

Supplemental Figures and Legends

Figure S2 - Normal motor skills, cognitive functions, and social behaviors in astroglial Sox2 deficient mice (related to Figure 1)

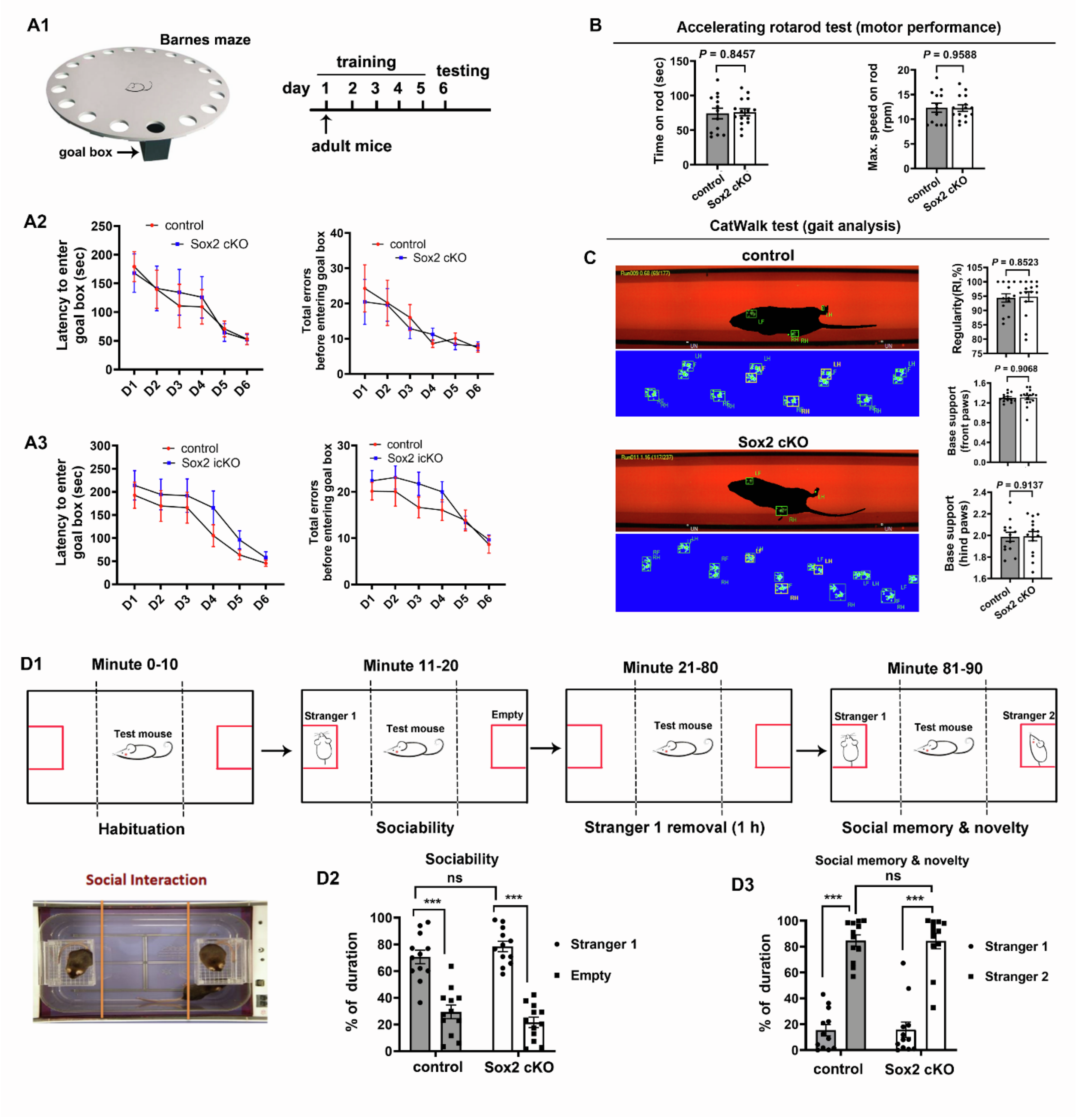

(A) Cognitive function evaluated by Barnes maze. (A1) Adult (P60) *Sox2* mutant animals (*Sox2* cKO and *Sox2* icKO) and respective littermate control mice were trained for 5 consecutive days (D1-D5) on Barnes maze for learning and memorizing the goal box with visual cue assistance followed by testing at day 6 (D6). Time latency to entering the goal box and total errors of non-goal box visits before entering the goal box were recorded for analysis in (A2) and (A3). Statistics: (A2) Latency: two-way ANOVA, $F_{(1,\ 108)}$ =

0.05092, $P = 0.8219$ for genotype; $F_{(5, 108)} = 4.573$, $P = 0.0008$ for time-course; total errors two-way ANOVA, $F_{(1, 108)} = 0.2035$, $P = 0.6528$ genotype; $F_{(5, 108)} = 4.930$, $P = 0.0004$ time-course. n = 11 *Sox2* cKO, 9 control. (A3) Latency two-way ANOVA, $F_{(1, 120)} = 3.474$, $P = 0.0648$ for genotype; $F_{(5, 120)} = 9.939$, $P < 0.0001$ for time-course; total errors, two-way ANOVA, $F_{(1, 120)} = 3.835$, $P = 0.0525$ for genotype; $F_{(5, 120)} = 9.840$, $P < 0.0001$ for time-course. n = 11 *Sox2* cKO, 11 control. *Sox2* icKO control mice were injected with tamoxifen at P14-P16 and tested on P60.

(B) Accelerating rotarod test of time on rod, two-tailed Student's t test, $t_{(26)} = 0.1966$, and maximal rotarod speed at the time of falling off, two-tailed Student's t test, $t_{(26)} = 0.0522$. n = 15 *Sox2* cKO, 13 control.

(C) CatWalk gait analysis by the automatic CatWalk XT equipment (Noldus). Representative images show footprints and gaits in unforced moving of *Sox2* cKO and control mice. Gait regularity index is the percentage of regular paw placements relative to the total irregular and regular paw placements. Two-tailed Student's *t* test, $t_{(26)} = 0.1880$. Base of support of front paws is assessed by the distance (cm) of both forelimbs to each other. Two-tailed Student's t test, $t_{(26)} = 0.1182$. Base of support of hind paws is assessed by the distance (cm) of both hindlimbs to each other. Two-tailed Student's t test, $t_{(26)} = 0.1094$. n = 15 *Sox2* cKO, 13 control.

(D) Three-chamber social interaction test. (D1) Schematic procedures of mouse social interaction assessed by three-chamber test. Both *Sox2* cKO and littermate control mice were tested at 2 months old. (D2) Sociability. Two-way ANOVA, $F_{(1, 44)} = 3.374e\text{-}006$, $P > 0.9985$ for genotype; $F_{(1, 44)} = 116.7$ $P < 0.0001$ for animals. (D3) Short-term social memory. Two-way ANOVA. $F_{(1, 44)} = 5.572e\text{-}006$, $P = 0.9981$ for genotype; $F_{(1, 44)} = 169.0$, $P < 0.0001$ for animals. n = 12 *Sox2* cKO, 12 control. Tukey's multiple comparison test, ns, $P > 0.05$, *** $P < 0.001$.

Supplemental Figures and Legends

Figure S3 - Astrocytic *Sox2* deletion perturbs astroglial molecular differentiation in the postnatal CNS (related to Figure 2)

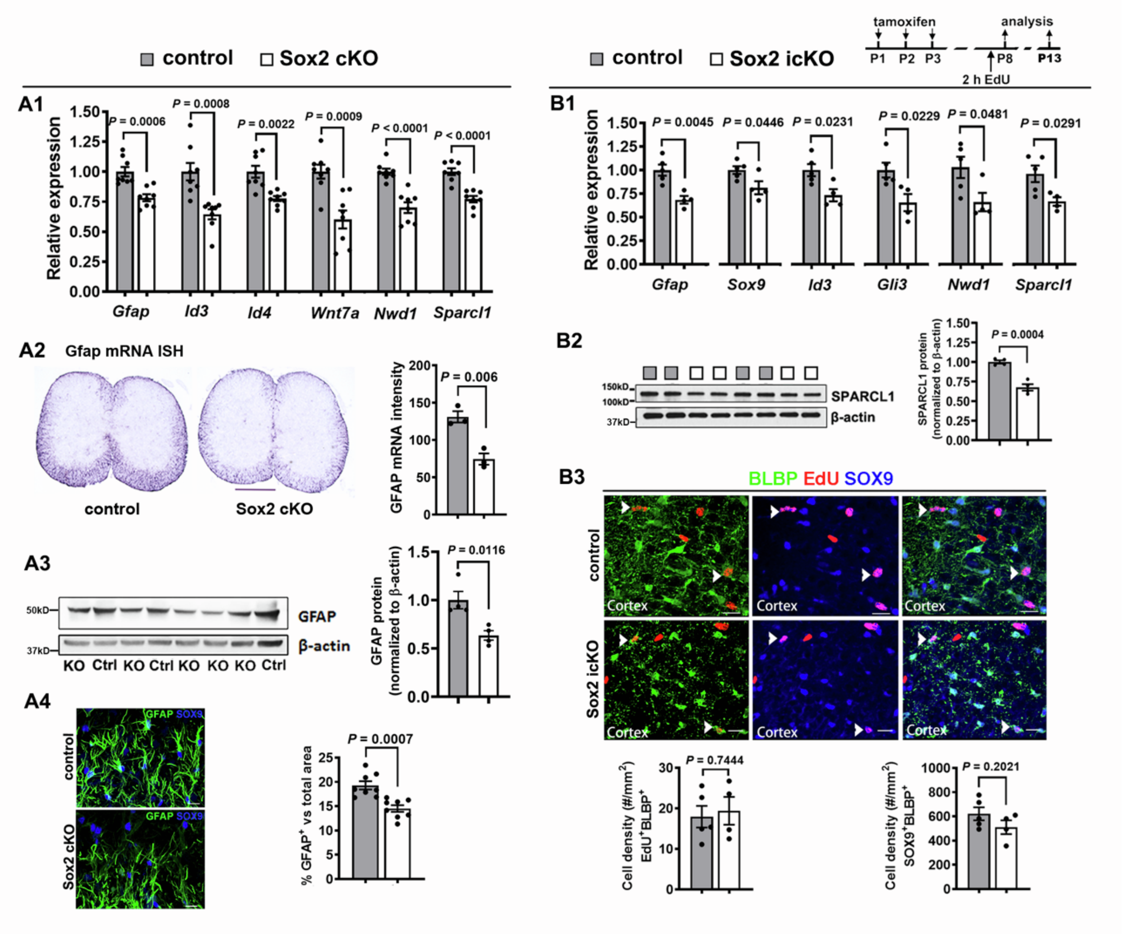

(A1) RT-qPCR assay of astrocyte-enriched genes. Two-tailed Student's t test, $t_{(14)}$ = 4.448 *Gfap*, $t_{(14)}$ = 4.228 *Id3*, $t_{(14)}$ = 4.181 *Id4*, $t_{(14)}$ = 4.220 *Wnt7a*, $t_{(14)}$ = 5.826 *Nwd1*, and $t_{(14)}$ = 6.013 *Sparcl1*.

(A2) *Gfap* mRNA in situ hybridization (ISH) and quantification. Two-tailed Student's t test, $t_{(4)}$ = 5.313.

(A3) Western blot and quantification of GFAP protein level. Two-tailed Student's t test, $t_{(6)}$ = 3.584.

(A4) Representative images of GFAP and astrocyte nuclear marker SOX9 and the percentage of GFAP$^+$ area among total assessed area. Two-tailed Student's t test, $t_{(14)}$ = 4.348. Scale bar = 10 µm. Data in panels A1-A4 were collected from P14 spinal cord. Scale bar, 10 µm.

(B1) Expression of astrocyte-enriched genes in the forebrain of P13 mice (tamoxifen at P1-P3). Two-tailed Student's t test, $t_{(7)}$ = 4.110 *Gfap*, $t_{(7)}$ = 2.440 *Sox9*, $t_{(7)}$ = 2.896 *Id3*, $t_{(7)}$ = 2.902 *Gli3*, $t_{(7)}$ = 2.391 *Nwd1*, and $t_{(7)}$ = 2.736 *Sparcl1.*

Supplemental Figures and Legends

(B2) Western blot images and quantification of mature astrocyte enriched protein SPARCL1 in the forebrain of P13 mice (tamoxifen at P1-P3). Two-tailed Student's t test, $t_{(6)} = 6.996$.

(B3) Representative confocal images and quantifications of BLBP, SOX9, and EdU (2 hr pulse labeling) immunostaining in the cerebral cortex of P8 mice (tamoxifen at P1-P3). Arrowheads point to triple positive astrocytes. Scale bar = 20 um. Two-tailed Student's t test, $t_{(7)} = 0.3392$, density of $EdU^{+}BLBP^{+}$ proliferating astrocytes; $t_{(7)} = 1.407$ cerebral cortex, density of $SOX9^{+}BLBP^{+}$ astrocytes. Scale bar, 20 μm.

Figure S4 – Isolation and culture of primary resting astrocytes in serum-free medium (related to Figure 3 and 4)

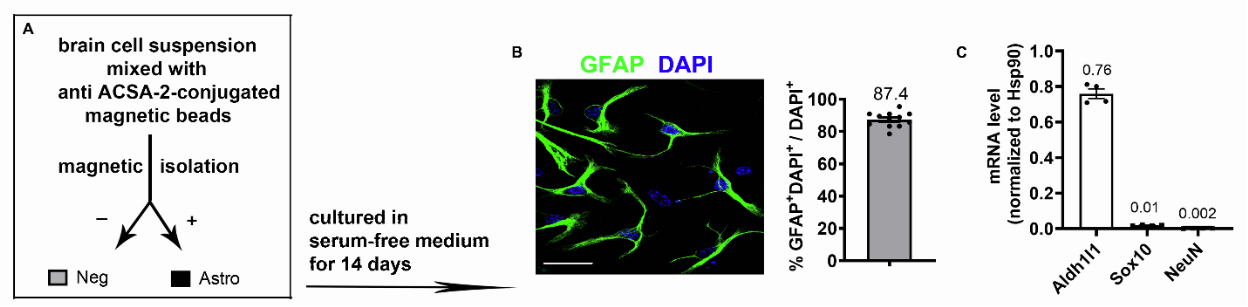

(A) Diagram of primary astrocyte isolation using MACS MicroBead Technology. Brain cell suspension was mixed with anti ACSA-2-conjugated magnetic beads, and loaded on to a MACS Column, which is placed in the magnetic field of a MACS Separator. The ACSA-2⁺ cells are retained within the column. The unlabeled cells run through and collected as Neg part, which is deleted of ACSA-2⁺ cells. After removing the column from the magnetic separator, ACSA-2⁺ cells were eluted in serum-free medium, seeded in culture dishes, and cultured for 14 days in vitro.

(B) Representative confocal image of primary astrocyte cultured for 14 days stained with GFAP and DAPI. Percentage of $GFAP^{+}/DAPI^{+}$ cells within the total DAPI population show that 87.4% of cells are $GFAP^{+}$ astrocytes in the culture for 14 days. Scale bar, 50 μm.

(C) RT-qPCR assay of astroglial lineage marker *Aldh1l1*, oligodendroglial lineage marker *Sox10* and neuronal lineage marker *NeuN* in the primary culture, demonstrating the purity of astrocyte and absence of oligodendrocytes and neurons.
